# Evolutionarily Optimal Risk Aversion

**DOI:** 10.1111/risa.70250

**Published:** 2026-04-19

**Authors:** Chmura, Nguyen, Biermann

**Affiliations:** ^1^ Nottingham Business School Nottingham Trent University Nottingham UK; ^2^ School of Economics University of Sheffield Sheffield UK

**Keywords:** risky choice, risk attitudes, evolution of preferences, HS&E

## Abstract

In an experimental choice situation, we identify risk‐acceptability thresholds and show how such thresholds are updated in response to benchmark information, a recurrent feature of health, safety, and environmental (HS&E) risk governance. We present a theoretical framework linking the observed behavior to an underlying evolutionary parameter, which in this case is (an abstract notion of) risk aversion. The theoretical model allows to predict how the experimental subjects adjust their risk aversion when informed about the risky choices of others. Applications of the framework arise naturally in HS&E settings, where individuals and organizations revise risk thresholds by observing peers, experienced coworkers, or acknowledged experts. By distinguishing confidence‐driven inertia from trust‐driven overreaction, the paper provides actionable guidance for HS&E risk communication and adaptive risk management.

## 1 Introduction

In evolutionary settings, the maximization of the number of surviving offspring is the ultimate objective which even preferences have to serve (see, e.g., Lande and Arnold 1985; Krebs 2009; Breslin 2013; Portera and Bartalesi 2016).^1^ This article formalizes and empirically tests the idea that preferences of people are associated with evolutionarily relevant parameters. Preferences are therefore not exogenous but result from a “hard‐wired” drive of a decision maker to get as close as possible to an optimal value of the evolutionary parameter. For example, the degrees of *compassion*, *patience*, or *aggression* may matter evolutionarily in different situations, and the observed preferences of a decision maker may follow downstream of a more fundamental desire to reach the optimal value of the evolutionary parameter, that is, to be compassionate, patient, or aggressive at an evolutionarily optimal level.

This paper has theoretical and practical‐empirical contributions. On a theoretical level, we model a decision maker for whom the adopted preferences depend on an underlying continuous parameter. We introduce the concept of a *thrive function*, which is a mapping M of an evolutionary parameter into the set of preferences. If $r^{*}$ is the evolutionarily optimal value of that parameter, then $M(r^{*})$ are those preferences of the decision maker that would maximize her evolutionary outcome. We will identify properties a thrive function should fulfill in order for the preferences displayed by the decision maker to be linked in a logical way with the underlying parameter.

As a practical and empirical contribution, we analyze *risky choice* using the theoretical framework of our model. In this context, the underlying evolutionary parameter is *risk aversion*.^2^ Throughout evolutionary history, the outcomes of risky situations were gains or losses in resources that could be used for mate search and kin feeding, and many such situations involved a possibility of injury or death (bringing further reproduction to an abrupt end). Therefore, the degree of risk aversion adopted in such critical situations had an impact on a human's survival and reproduction potential. As an illustration, consider distant ancestors in hunting or conflict situations. If they were reckless, they could perish in the pursuit of killing animals or in a fight with other group members, in which case their expected offspring was less numerous than if they had adopted a more cautious approach. On the other hand, reluctance to take risks would entail feeble hunting success and giving away too much to others, leading to less resources and a potential loss of status. This illustrates that by the expected offspring criterion, it is both possible to be too risk‐averse and too risk‐loving in a given situation, implying that there should be a context‐dependent *evolutionarily optimal* degree of risk aversion between the extremes.

When applied to risk preferences, our model yields empirically testable predictions about how the choice behavior of humans will be affected through learning about the *choices of others*. The model predicts that knowledge about the choices of others may lead to preference changes, resulting from agents updating their original opinions about what is the evolutionarily optimal risk attitude in the situation at hand. Specifically, it follows that the extent to which decision makers adjust their preferences depends on the confidence they have in their own judgment versus their confidence in the judgments of others. Based on these variables, we can, among other testable hypotheses, predict who will modify their preferences upon observing others and who will not. We test these predictions in a sequential lottery choice experiment devised for this purpose, and we find affirmative evidence.

Modern health, safety, and environmental (HS&E) contexts yield ample applications of our theory as individuals frequently adjust their willingness to accept health risks, workplace hazards, or environmental exposures in response to perceived threats, social information, and protective norms (see the discussions, e.g., in Kasperson et al. 1988; Dosman et al. 2001; Lindell and Hwang 2008; Ho et al. 2022). We will argue that some of the risk attitude changes observed in HS&E contexts reflect—on a fundamental level—the adaptive pressure to balance caution and risk‐taking in the pursuit of maximizing the expected number of offspring, as hypothesized in this paper.

As illustrations, consider how vaccine uptake (for Covid and other viruses) is influenced by the behaviors of colleagues, neighbors, and friends (e.g., LaJoie et al. 2018; Patelarou et al. 2022; McCready et al. 2023), or how workers in hazardous occupations modify their safety practices when they observe colleagues either taking precautions or ignoring them (Wakefield et al. 2010). Individuals may initially underestimate or overestimate the health or safety risks they face, but their risk‐taking behaviors changes rapidly when they observe the behavior of others. A worker who normally ignores protective equipment may become more cautious after seeing peers consistently adopt safety measures, whereas someone observing widespread rule‐breaking may become less risk‐averse (Dean 2014; Maglio et al. 2016; Wong et al. 2020). This mirrors the evolutionary logic underlying our model: natural selection would favor individuals who adjust their risk attitudes flexibly, drawing on both personal judgment and social information about what constitutes an advantageous level of risk‐taking under prevailing conditions.

We offer this paper for publication in the special issue of *Risk Analysis* in commemoration of Daniel Kahneman (1934–2024). While not at the center of *Prospect Theory*, the question of what causes preferences to change was a topic that he was always interested in. The phenomenon of *preference reversal* had been described by Lichtenstein and Slovic (1971), triggering a protracted debate about the reasons why this would happen. Kahneman joined his friends Amos Tversky and Paul Slovic relatively late in this debate in order to contribute an important (some would say: authoritative) explanation for this phenomenon (Tversky et al. 1990). Likewise, a prominent brainchild of Kahneman were *framing effects*. From the beginning, these were shown to have an impact on choice problems involving *lotteries*, where they caused people to *change their preferences*, as in the original paper of Tversky and Kahneman (1981). As our experiment demonstrates how (risk) preferences change, we believe that Daniel Kahneman would have been interested in our empirical findings—but would he have liked the evolutionary story?

While he was not an evolutionary psychologist in a strict sense, Kahneman was no stranger to evolutionary arguments in support of his behavioral observations. For example, he attributed *loss aversion* in Prospect Theory to evolutionary pressures: “When directly compared or weighted against each other, losses loom larger than gains. This asymmetry between the power of positive and negative expectations or experiences has an evolutionary history. Organisms that treat threats as more urgent than opportunities have a better chance to survive and reproduce” (Kahneman 2011). Against this backdrop, we believe that he would have been open‐minded to consider the theoretical explanation we offer.

This paper contributes to the analysis of risk‐related decision‐making with a particular focus on HS&E contexts. We develop a formal decision‐theoretic model in which observed choices reflect the adoption and updating of *risk‐acceptability thresholds* in response to social and benchmark information, a central feature of HS&E risk governance. The model is tested experimentally using a sequential choice design that elicits how individuals adjust their risk thresholds when informed about the risky choices of others. The results reveal systematic heterogeneity in updating behavior that is related to proxies for confidence in one's own judgment and trust in others, helping to explain why identical risk signals can lead to underadjustment in some settings and overadjustment in others. We further illustrate the relevance of the framework through applications to real‐world HS&E decision problems, including the escalation of safety thresholds following near‐miss events, responses to public health guidance, and the formation of environmental risk acceptability, thereby demonstrating how the model informs risk communication, organizational learning, and adaptive risk management.

The remainder of the paper is structured as follows. Section 2 presents a rigorous mathematical model on how the adoption of preferences can be linked to a continuous evolutionary parameter. Section 3 uses this framework to predict what will happen when agents observe the choices of *others*. Section 4 features a description of the experiment we have conducted and states hypotheses on the experimental results we expect to find. In Section 5, we present and interpret the experimental outcomes in view of our hypotheses. Section 6 offers a general discussion of how our theory, for example, how it squares with the well‐known finding of *risky shift* (Wallach et al. 1962). We also consider what it means that one of our hypotheses was rejected in the experiment, and we respond to some potential objections against the assumptions of our model. Section 7 zooms in on the relevance of our findings in HS&E settings, and Section 8 concludes the paper. The proofs of the mathematical results can be found in Appendix A and a description of the experiment in Appendix B.

## 2 The Thrive Function

This section presents a formal decision‐theoretic model in which the preferences adopted by the decision maker depend on a continuous parameter. This is motivated by our hypothesis that in choice situations with evolutionary significance, preferences are not exogenous but follow from deciding on an evolutionarily relevant value. The decision maker's displayed preferences are then endogenously determined by that prior choice.

When describing our model, we will call the elements of the choice set *lotteries* and the parameter the *degree of risk aversion*, but this is not the only possible interpretation. In other potential applications of this framework, the choice set could have other objects, and the evolutionary parameter could have different interpretations, for example, it could be the level of *aggression*, the level of *patience*, or the level of *compassion* inherent in the agent's choices.

Technically, our agent has to adopt preferences over a finite set L. By *preferences*, we mean a strict total relation ≻ over L.^3^ The set of all strict total relations over L is denoted by P.

We assume that in a given decision problem, any preferences that can be adopted are associated with an evolutionary parameter $r\in (\underline{r},\bar{r})\subset R$.^4^

The evolutionary significance of adopted preferences is formalized through a function $M:(\underline{r},\bar{r})\to \;P$ that assigns an element ${≻}_{r}\;\in \;P$ to each $r\in (\underline{r},\bar{r})$, with the interpretation that ${≻}_{r}$ are the preferences that are evolutionarily most beneficial if r was the optimal evolutionary value in the decision situation at hand. We call M a *thrive function*.^5^

To simplify the analysis, we remove some elements from L that are fundamentally irrelevant but have the potential to complicate matters:

Definition 1 (Domination) If there are two lotteries $l^{\prime },l^{\prime \prime }\in L$ such that $l^{\prime }{≻}_{r}l^{\prime \prime }$ for all $r\in (\underline{r},\bar{r})$, then $l^{\prime \prime }$ is called a *dominated* lottery.

When a lottery $l^{\prime \prime }$ is dominated, it would *never* be chosen by a decision maker who optimizes her evolutionary outcome, regardless of what she considers to be the correct value of r in the situation at hand. By an argument resembling *Independence of Irrelevant Alternatives* (Arrow 1951), removing $l^{\prime \prime }$ from L would not change the agent's actual choice whenever the other elements in L remain on offer. This simplifies some of our arguments, and we therefore assume for this analysis that there are no dominated lotteries in L.

A thrive function M can have the *single‐crossing property* (Apesteguia et al. 2017; Chiappori et al. 2019; Barseghyan et al. 2021), which in the context of this model can be motivated as follows. Consider a decision maker who believes that the optimal level of risk aversion in the situation is $r^{\prime }$, and assume that according to the preferences ${≻}_{r^{\prime }}$, she prefers lottery $l^{\prime }$ to lottery $l^{\prime \prime }$, that is, $l^{\prime }{≻}_{r^{\prime }}l^{\prime \prime }$. Later, she updates her belief, maybe because she obtained new information about some characteristic of the situation, and now she feels that the optimal risk aversion is $r^{\prime \prime }>r^{\prime }$. Accordingly, her preferences change to ${≻}_{r^{\prime \prime }}$. If under these new preferences, she would prefer $l^{\prime \prime }$ to $l^{\prime }$, that is, $l^{\prime \prime }{≻}_{r^{\prime \prime }}l^{\prime }$, it would suggest that $l^{\prime }$ is, in some sense, *riskier* than $l^{\prime \prime }$ because as her risk aversion increases, her choice switches from $l^{\prime }$ to $l^{\prime \prime }$. Consequently, it would be reasonable to expect that for an even *higher* risk aversion $r^{\prime \prime \prime }>r^{\prime \prime }$, she would *keep* to preferring $l^{\prime \prime }$ to $l^{\prime }$, that is, $l^{\prime \prime }{≻}_{r^{\prime \prime \prime }}l^{\prime }$. By the same logic, if she initially prefers $l^{\prime }$ to $l^{\prime \prime }$ but reverses her preferences due to a *reduction* of risk aversion, her preferences should not switch again if her risk aversion decreases further. This idea is captured in:

Property 1 (Single‐crossing property) Let $l^{\prime },l^{\prime \prime }\in L$ and $r^{\prime }<r^{\prime \prime }$. If M is single‐crossing, then from $l^{\prime }{≻}_{r^{\prime }}l^{\prime \prime }$ and $l^{\prime \prime }{≻}_{r^{\prime \prime }}l^{\prime }$ follows $l^{\prime }{≻}_{r}l^{\prime \prime }$ for all $r<r^{\prime }$ and $l^{\prime \prime }{≻}_{r}l^{\prime }$ for all $r>r^{\prime \prime }$.

Lemma 1 Assume that M is single‐crossing and there are no dominated lotteries in L. Then, for $l^{\prime },l^{\prime \prime }\in L$ with $l^{\prime }\neq l^{\prime \prime }$, exactly one of the following statements is true:

1. There exists a value $r\left(l^{\prime },l^{\prime \prime }\right)\in \left(\underline{r},\bar{r}\right)$ such that $l^{\prime }{≻}_{r}l^{\prime \prime }$ if $r<r(l^{\prime },l^{\prime \prime })$ and $l^{\prime \prime }{≻}_{r}l^{\prime }$ if $r>r(l^{\prime },l^{\prime \prime })$.
2. There exists a value $r\left(l^{\prime \prime },l^{\prime }\right)\in \left(\underline{r},\bar{r}\right)$ such that $l^{\prime \prime }{≻}_{r}l^{\prime }$ if $r<r(l^{\prime \prime },l^{\prime })$ and $l^{\prime }{≻}_{r}l^{\prime \prime }$ if $r>r(l^{\prime \prime },l^{\prime })$.

Note that we do not specify whether $l^{\prime }{≻}_{r(l^{\prime },l^{\prime \prime })}l^{\prime \prime }$ or $l^{\prime \prime }{≻}_{r(l^{\prime },l^{\prime \prime })}l^{\prime }$. For our analysis, it does not matter which of the two is the case. Likewise, it does not matter whether $l^{\prime }{≻}_{r(l^{\prime \prime },l^{\prime })}l^{\prime \prime }$ or $l^{\prime \prime }{≻}_{r(l^{\prime \prime },l^{\prime })}l^{\prime }$.^6^

For our next steps, we need to define a metric on L. In line with straightforward intuition, we will say that the more comparisons one agents makes differently than another agent, the more remote are their preferences. Formally, we define:

(1) $$d\left({≻}^{\prime },{≻}^{\prime \prime }\right):=\frac{1}{2}|\{\left(l^{\prime },l^{\prime \prime }\right)\in L\times L\;|\;l^{\prime }{≻}^{\prime }l^{\prime \prime }\wedge l^{\prime \prime }{≻}^{\prime \prime }l^{\prime }\}|.$$

There are $d({≻}^{\prime },{≻}^{\prime \prime })$ binary comparisons between lotteries in L that are made differently by ${≻}^{\prime }$ and ${≻}^{\prime \prime }$. We multiply the number of different pairs by 1/2 as otherwise, $\left(l^{\prime },l^{\prime \prime }\right)$ and $\left(l^{\prime \prime },l^{\prime }\right)$ would both be counted for the same lotteries $l^{\prime },l^{\prime \prime }$ even though it is the same comparison.^7^

Lemma 2 The function d:L×L→N∪{0} defined by (1) is a metric on L.

Technically, the above metric is a two‐dimensional version of a *Hamming metric* (Hamming 1950).^8^  9

The metric d(·,·) allows us to define another intuitive property a thrive function can have:

Property 2 (Linearity) Assume $r^{\prime }<r^{\prime \prime }$ with $d({≻}_{r^{\prime }},{≻}_{r^{\prime \prime }})>1$. If M is linear, there are values $r^{1},r^{2}$ with $r^{\prime }\leq r^{1}<r^{2}\leq r^{\prime \prime }$ such that for all $r\in (r^{1},r^{2})$, it holds $d(r,r^{\prime \prime })=1$.^10^

Linearity means that if there is a distance greater than one between two relations ${≻}_{r^{\prime }}$ and ${≻}_{r^{\prime \prime }}$ in the image of M, then we can find a range of r between $r^{\prime }$ and $r^{\prime \prime }$ where the distance to ${≻}_{r^{\prime \prime }}$ is exactly one. We consider this to be an intuitive property for many applications of this model, including the one where r is interpreted as risk aversion: linearity implies that there exists always a change in the value of r that is so small that the resulting change of the adopted preferences, if any, will also be minimal, meaning that it triggers only two lotteries to be swapped in the ranking. When r ranges over $\left(\underline{r},\bar{r}\right)$, the function values ${≻}_{r}$ of a linear thrive function will not “jump” in terms of their distance from a given element in P. Assume that r initially stands at $r^{\prime }$ and is mapped into ${≻}_{r^{\prime }}$. If r increases or decreases, the next relation into which r is mapped will have a distance of *one* from ${≻}_{r^{\prime }}$, the subsequent one a distance of *two*, and so on. We assume from here onward that the decision maker's thrive function M is linear.

Linearity ensures that the values defined in Lemma 1 are distinct:

Lemma 3 Let $\{l^{1},l^{2},l^{3},l^{4}\}\in L$ with $\left\{l^{1},l^{2}\right\}\neq \left\{l^{3},l^{4}\right\}$. If the thrive function M is single‐crossing and linear, then $r\left(l^{1},l^{2}\right)\neq r\left(l^{3},l^{4}\right)$.

Proposition 1 If the function M is single‐crossing and linear, then $\left(\underline{r},\bar{r}\right)$ can be partitioned into n intervals $I_{1},\text{…},I_{n}$ (each of which may be open, closed, or semiclosed) such that:

1. ${≻}_{r^{\prime }}\;=\;{≻}_{r^{\prime \prime }}$ if and only if $r^{\prime },r^{\prime \prime }\in I_{j}$ for one j∈{1,…,n}.
2. The n intervals can be labeled in a way that fulfils:
$$r^{\prime }\in I_{i},r^{\prime \prime }\in I_{j}\Rightarrow d\left({≻}_{r^{\prime }},{≻}_{r^{\prime \prime }}\right)=\left|i-j\right|.$$

The above result provides conditions on when it is possible to rank preference relations associated with an underlying parameter such that: (a) higher ranked preferences correspond to higher parameter values and (b) neighboring preferences differ by exactly one comparison. An example where this is relevant is the menu of lotteries of Holt and Laury (2002), which has been used in countless experiments to establish the risk attitudes of experimental subjects^11^ and is also deployed in our own experiment (see Section 4).

The lotteries in Holt and Laury (2002) are arranged in two groups A and B. There are 10 lotteries in A and 10 lotteries in B, and the payoffs of all lotteries in each group are identical, being $2 and $1.60 in Group A, and $3.85 and $0.10 in Group B. Within each group, the lotteries just differ by their probabilities. Denoting by p the probability of obtaining the first payoff, there is exactly one lottery in each group with p=0.1,p=0.2,…,p=0.9, and p=1. In setbuilder notation, we can write the two sets of lotteries as

$$A:=\left\{\;[\;p,1-p\;|\;\$2,\$1.60\;]\;|\;p\in \{0.1,0.2,\text{…},0.9,1\}\;\right\}$$

and

$$B:=\left\{\;[\;p,1-p\;|\;\$3.85,\$0.1\;]\;|\;p\in \{0.1,0.2,\text{…},0.9,1\}\;\right\}.$$

In experiments making use of this menu, subject are typically asked (explicitly or implicitly) for which probabilities they prefer the lotteries in Group A, and for which probabilities they prefer the lotteries in Group B. If the decision makers have a Constant Relative Risk Aversion (CRRA) utility function (Arrow 1974; Wakker 2008),^12^ there will be a unique *switching point* where they move from the lotteries in Group A to the lotteries in Group B. For example, for p≤0.4, the decision maker might prefer the lotteries in set A, but if p≥0.5, she switches to set B. In this case, the switching point, defined as the highest scenario in Table 3 where *Option* A is preferred, would be 4. Table 1 shows the approximate switching points as a function of the underlying risk aversion r, using the exact utility function U(x) stated in footnote (not a transformation thereof).

**TABLE 1:** How relative risk aversion r determines the switching points in Table 3.

Value of r	Switching point (= highest scenario in Table 3 where *Option* A is preferred)
0≤r≤0.1	4
0.1<r≤0.3	5
0.3<r≤0.5	6
0.5<r≤0.95	7
0.95<r≤1.2	8
1.2<r	9 and 10

**TABLE 2:** Examples of belief updating for λ=0.3,λ=0.5, and λ=0.9.

λ=0.3	${≻}_{1}$	${≻}_{2}$	${≻}_{3}$	${≻}_{4}$	${≻}_{5}$	${≻}_{6}$	${≻}_{7}$	${≻}_{8}$	${≻}_{9}$	${≻}_{10}$	${≻}_{11}$	Minimizer
Probabilities *p*	0.07	0.00	0.12	0.19	0.04	0.05	0.01	0.21	0.03	0.14	0.14	8
Expected distance	5.69	4.82	3.97	3.34	3.10	2.92	2.85	2.80	3.17	3.60	4.31	
Probabilities *q*	0.17	0.15	0.08	0.10	0.08	0.02	0.01	0.03	0.11	0.08	0.16	4
Expected distance	4.52	3.87	3.53	3.35	3.36	3.54	3.74	3.98	4.28	4.80	5.48	
Probabilities *s*	0.10	0.05	0.10	0.16	0.05	0.04	0.01	0.16	0.06	0.12	0.15	6
Expected distance	5.34	4.54	3.84	3.34	3.18	3.11	3.12	3.15	3.50	3.96	4.66	
λ=0.5	${≻}_{1}$	${≻}_{2}$	${≻}_{3}$	${≻}_{4}$	${≻}_{5}$	${≻}_{6}$	${≻}_{7}$	${≻}_{8}$	${≻}_{9}$	${≻}_{10}$	${≻}_{11}$	Minimizer
Probabilities *p*	0.03	0.20	0.27	0.02	0.15	0.03	0.13	0.03	0.01	0.11	0.01	3
Expected distance	3.76	2.82	2.28	2.29	2.34	2.70	3.11	3.77	4.50	5.26	6.24	
Probabilities *q*	0.01	0.03	0.13	0.13	0.13	0.13	0.07	0.06	0.08	0.11	0.11	6
Expected distance	5.54	4.56	3.64	2.97	2.56	2.41	2.52	2.77	3.14	3.69	4.46	
Probabilities *s*	0.02	0.12	0.20	0.08	0.14	0.08	0.10	0.05	0.05	0.11	0.06	5
Expected distance	4.65	3.69	2.96	2.63	2.45	2.55	2.81	3.27	3.82	4.47	5.35	
λ=0.9	${≻}_{1}$	${≻}_{2}$	${≻}_{3}$	${≻}_{4}$	${≻}_{5}$	${≻}_{6}$	${≻}_{7}$	${≻}_{8}$	${≻}_{9}$	${≻}_{10}$	${≻}_{11}$	Minimizer
Probabilities *p*	0.01	0.03	0.08	0.03	0.00	0.11	0.18	0.06	0.20	0.19	0.11	9
Expected distance	6.74	5.77	4.85	4.08	3.38	2.68	2.20	2.07	2.07	2.48	3.26	
Probabilities *q*	0.08	0.18	0.07	0.00	0.17	0.09	0.18	0.10	0.00	0.03	0.09	5
Expected distance	4.44	3.61	3.12	2.79	2.46	2.47	2.67	3.22	3.98	4.74	5.56	
Probabilities *s*	0.07	0.16	0.07	0.01	0.15	0.09	0.18	0.10	0.02	0.04	0.09	6
Expected distance	4.67	3.82	3.30	2.92	2.55	2.49	2.62	3.11	3.79	4.51	5.33	

**TABLE 3:** The choice task given to the experimental subjects.

Scenario #	Asset A	Asset B
1	10% chance of 20 ECU and 90% chance of 16 ECU	10% chance of 38.5 ECU and 90% chance of 1 ECU
2	20% chance of 20 ECU and 80% chance of 16 ECU	20% chance of 38.5 ECU and 80% chance of 1 ECU
3	30% chance of 20 ECU and 70% chance of 16 ECU	30% chance of 38.5 ECU and 70% chance of 1 ECU
4	40% chance of 20 ECU and 60% chance of 16 ECU	40% chance of 38.5 ECU and 60% chance of 1 ECU
5	50% chance of 20 ECU and 50% chance of 16 ECU	50% chance of 38.5 ECU and 50% chance of 1 ECU
6	60% chance of 20 ECU and 40% chance of 16 ECU	60% chance of 38.5 ECU and 40% chance of 1 ECU
7	70% chance of 20 ECU and 30% chance of 16 ECU	70% chance of 38.5 ECU and 30% chance of 1 ECU
8	80% chance of 20 ECU and 20% chance of 16 ECU	80% chance of 38.5 ECU and 20% chance of 1 ECU
9	90% chance of 20 ECU and 10% chance of 16 ECU	90% chance of 38.5 ECU and 10% chance of 1 ECU
10	100% chance of 20 ECU	100% chance of 38.5 ECU
**Sw. point #**	**Subjects had to choose one of the following alternatives**:	
0	Choose Asset B for ALL scenarios	
1	Choose Asset A for Scenario 1 and Asset B for Scenario 2 to 10	
2	Choose Asset A for Scenario 1 to 2 and Asset B for Scenario 3 to 10	
3	Choose Asset A for Scenario 1 to 3 and Asset B for Scenario 4 to 10	
4	Choose Asset A for Scenario 1 to 4 and Asset B for Scenario 5 to 10	
5	Choose Asset A for Scenario 1 to 5 and Asset B for Scenario 6 to 10	
6	Choose Asset A for Scenario 1 to 6 and Asset B for Scenario 7 to 10	
7	Choose Asset A for Scenario 1 to 7 and Asset B for Scenario 8 to 10	
8	Choose Asset A for Scenario 1 to 8 and Asset B for Scenario 9 to 10	
9	Choose Asset A for Scenario 1 to 9 and Asset B for Scenario 10	
10	Choose Asset A for ALL Scenarios	

Every switching point is associated with a unique preference relation over the set of 20 lotteries that are included in the table. To illustrate this point, label the A‐lotteries $A_{1},\text{…},A_{10}$ with increasing probability p, and the B‐lotteries $B_{1},\text{…},B_{10}$ with increasing probability p (see the “scenarios” in Table 3). If, for example, the switching point is 5, then we have 0.1<r≤0.3. From here, we infer that $A_{4}≻B_{4}$, $A_{3}≻B_{3}$, $A_{2}≻B_{2}$, and $A_{1}≻B_{1}$, as well as all other comparisons, by computing the expected utility of the lotteries with the utility function given in footnote, setting r to be a value in the specified interval.^13^  14

As can be seen, implicit in the Holt and Laury (2002) menu of lotteries is a continuous variable r that is mapped into a set of preference relations over those lotteries. Moreover, there is an interval structure of r, having the same properties as the interval structure in Proposition 1. To illustrate the last point, consider Table 3, where the “scenarios” in the upper half of the table are the lotteries of Holt and Laury (2002).^15^ If someone chooses Asset A in scenarios 1–3, and Asset B in all other scenarios, her switching point is 3. If another decision maker has switching Point 4, then the preferences of the two persons are different by exactly one comparison:^16^ they only rank the lotteries in scenario 4 differently, while all other scenarios comparisons are made in the same way.

When we considered to run an experiment based on the lottery menu of Holt and Laury (2002), we were not sure whether their connection between risk aversion and preferences can be generalized. Is it required to design the lotteries in the choice menu a special way so as to obtain an interval mapping from the degree of risk aversion into the set of preferences? Obviously, we did not want to develop a theory that only explains the very choice situation constructed by Holt and Laury (2002) but does not carry over to other lottery choice problems. Proposition 1 addresses that problem by stating sufficient conditions for a choice problem to have the same structure as the lottery menu of Holt and Laury (2002). If the connection between the underlying parameter and the preferences can be represented by a linear and single‐crossing thrive function, *any* choice set leads to a structure as in Holt and Laury (2002). This may be of relevance for other researchers concerned with the generalizability of results derived from the Holt and Laury (2002) lottery menu, and it may also be relevant in applications where r is not risk aversion but some other parameter.^17^

## 3 Observing Others' Choices

Up to this point, we have described how the decision maker forms preferences in response to a perceived optimal value of the evolutionary parameter r. However, we still need to formalize what it means to hold an opinion about the evolutionarily optimal level $r^{*}\in \left(\underline{r},\bar{r}\right)$, and, for making empirical predictions, we need to model how this opinion is affected by new information. This section is devoted to this aspect.

Importantly, we assume that when multiple decision makers are confronted with the same decision situation, they all have the same thrive function. Heterogeneity between the agents stems from different views on what is the optimal value of r, not from different views on the preferences that should be adopted for a *given* value of r. Specifically, in view of Lemma 1, all agents will agree that if the evolutionarily optimal value of r was lower than $r(l^{\prime },l^{\prime \prime })$, one should rank $l^{\prime }≻l^{\prime \prime }$, and if it was higher than $r(l^{\prime },l^{\prime \prime })$, one should rank $l^{\prime \prime }≻l^{\prime }$.

Our assumption makes sense if the individuals are sufficiently *similar*. For that case, an evolutionary argument can be made why in an evolutionary steady state, the thrive functions of all decision makers should be the same: if Agent A feels that for a given value r, the preferences ${≻}_{r}^{A}$ should be adopted, whereas Agent B feels that preferences ${≻}_{r}^{B}$ should be displayed, then if ${≻}_{r}^{A}\;\neq \;{≻}_{r}^{B}$, one of the two preferences would yield higher reproductive success in situations where the evolutionary optimal value was r, and agents who have the other preferences would, over the passage of many generations, eventually go extinct.^18^

We assume that the agent does not know with certainty what is the evolutionarily optimal level $r^{*}$ of the parameter but holds a belief $p:={\left\{p\left(i\right)\right\}}_{i\in Z}$ where p(i) for i∈{1,…,n} denotes the (subjective) probability of the event $r^{*}\in I_{i}$ (see Proposition 1). For technical reasons, it is convenient to define p(i) for *every* integer value i∈Z and set p(i)=0 if i∉{1,…,n}. We may construe the probability mass function p to be derived from an underlying continuous density function θ of $r^{*}$ over $\left(\underline{r},\bar{r}\right)$ by setting $p\left(i\right)=\int_{a}^{b}\theta \left(r\right)dr$, where a and b are the left and right boundaries of $I_{i}$.

Let $M(\underline{r},\bar{r})\subseteq P$ be the image of the thrive function M, and denote by ${≻}^{i}$ the element ${≻}_{r}\;\in M\left(\underline{r},\bar{r}\right)$ if $r\in I_{i}$. In view of the convention we made (p(i)=0 if i∉{1,…,n}) and the second statement of Proposition 1, we can express the expected distance of element ${≻}^{i}$ from the optimal relation ${≻}_{r^{*}}$ as

(2) $$E_{p}\left[d\left({≻}^{i},{≻}_{r^{*}}\right)\right]=\sum_{k=1,\text{…},\infty }k\left(p\left(i+k\right)+p\left(i-k\right)\right).$$

For k>0, the expression p(i+k)+p(i−k) in (2) is the probability under the belief p that ${≻}^{i}$ and ${≻}_{r^{*}}$ have a distance of k. $\sum_{k=1,\text{…},\infty }k\left(p\left(i+k\right)+p\left(i-k\right)\right)$ is thus the *expected* distance of ${≻}^{i}$ from ${≻}_{r}^{*}$.

We assume that a decision maker with belief p will adopt the preferences ${≻}^{i^{*}}\in M\left(\underline{r},\bar{r}\right)$ for which the expected distance to the evolutionarily optimal preferences is minimal, that is,

(3) $$i^{*}:=\underset{i\in \{1,\text{…},n\}}{arg min}E_{p}\left[d\left({≻}^{i},{≻}_{r^{*}}\right)\right].$$

It is well known that the median minimizes the expected absolute deviation from the realization of a random variable (see Lemma 4 below). Therefore, the median of the probability mass function p solves the minimization problem associated with (3). As is common in the literature (e.g., Mittelhammer 2012, Definition 3.1.1, p. 139), we define the median of a probability mass function $p:={\left\{p\left(i\right)\right\}}_{i=1,\text{…},n}$ to be m∈{1,…,n} such that both $\sum_{i=1,\text{…},m}p\left(i\right)\geq 0.5$ and $\sum_{i=m,\text{…},n}p\left(i\right)\geq 0.5$. Unless n is even and the values 1,…,n/2 have exactly the same joint probability mass as the values n/2+1,…,n, the median will be unique. While our model does not require a unique median, the possibility of multiple medians forces us to make awkward case distinctions throughout our remaining analyses without adding any additional qualitative insights. Pragmatically, we avoid these problems by making the simplifying assumption that all probability mass functions in our analyses have unique medians.^19^

Lemma 4 (Median minimizes expected absolute error) If m(p) is the unique median of the probability mass function p, then i=m(p) minimizes the expression

$$\sum_{k=1,\text{…},\infty }k\left(p\left(i+k\right)+p\left(i-k\right)\right).$$

Now assume that the agent with belief p learns about another belief q, with $q:={\left\{q\left(i\right)\right\}}_{i\in Z}$ where q(i) for i∈{1,…,n} denotes the subjective probability of the event $r^{*}\in I_{i}$. As before, we set q(i)=0 for i∉{1,…,n}. The origin of q could be the observation of another agent making choices compatible with preferences consistent with q, or it could simply be that our decision maker is told that other people have the belief q. Being confronted with q, the agent will update her own belief in accordance with a parameter λ∈[0,1], forming a new “synthesis belief” $s_{\lambda }$:

(4) $$s_{\lambda }={\left\{\lambda q\left(i\right)+\left(1-\lambda \right)p\left(i\right)\right\}}_{i\in Z}.$$

The parameter λ captures the agent's uncertainty about the accuracy of her own judgment as well as the trust she has in the reliability and trustworthiness of the belief q. If λ=0, finding out about q does not affect the agent's existing belief about the value of $r^{*}$ at all; her initial belief p is the same as her subsequent belief $s_{\lambda }$. This would be the extreme case where she has absolute confidence in her own perception, understanding, and expertise of the decision problem so that she dismisses any choice inconsistent with her initial belief p. On the other hand, if λ=1, she would fully adopt the belief q. If λ=0.5, she would put the same trust in her own judgment as well as in the judgment of whoever proposed q.

By Lemma 4, the median m(p)∈{1,…,n} of the random variable p solves the minimization problem (3). Accordingly, m(q) solves the analogous problem if the belief is q (i.e., $m\left(q\right):={arg min}_{i\in \{1,\text{…},n\}}E_{q}\left[d\left({≻}^{i},{≻}_{r^{*}}\right)\right]$), and $m(s_{\lambda })$ solves the problem if the belief is $s_{\lambda }$. With this notation, we state the following.

Lemma 5 Let p and q be probability mass functions defined on Z, supported on a finite set {1,…,n}⊂N, and let $s_{\lambda }={\left\{\lambda q\left(i\right)+\left(1-\lambda \right)p\left(i\right)\right\}}_{i\in Z}$ with λ∈[0,1] be a linear combination of p and q. If $m\left(q\right),m\left(p\right),m\left(s_{\lambda }\right)$ are the unique medians of the three functions and m(p)≤m(q), then $m\left(p\right)\leq m\left(s_{\lambda }\right)\leq m\left(q\right)$.

Our next and final result shows that the distance between ${≻}^{m(s_{\lambda })}$ and ${≻}^{m(q)}$ is weakly smaller than the distance between ${≻}^{m(p)}$ and ${≻}^{m(q)}$. This is, upon encountering a belief q, our agent, who previously adopted the preferences ${≻}^{m(p)}$, tends to *move closer toward* ${≻}^{m(q)}$.

Proposition 2 Let λ∈[0,1]. Then,

$$d\left({≻}^{m(s_{\lambda })},{≻}^{m(q)}\right)\leq d\left({≻}^{m(p)},{≻}^{m(q)}\right).$$

Table 2 illustrates this result with three examples for n=11 (this number was chosen because it corresponds with the experiment we will describe in the next section). Each example features two distributions p and q and their synthesis distribution $s_{\lambda }$ based on the values λ=0.3,λ=0.5, and λ=0.9. Under each preferences, we state the expected distance from the realization of the random variable $r^{*}$ if those preferences are adopted. The last column shows the medians of the distributions, which are the minimizers of the expected distances to $r^{*}$. For illustration, consider the first case with λ=0.3. Under belief p, the probability that $r^{*}\in I_{1}$, meaning that ${≻}_{1}$ are the optimal preferences, is 0.07. For $r^{*}\in I_{2}$, the probability is close to 0, and for $r^{*}\in I_{3}$, the probability is 0.12, and so forth. The respective expected distances from ${≻}_{r*}$ are 5.69, 4.82, and 3.97. The median of belief p is 8, which is the choice where the expected distance from ${≻}_{r*}$ is minimal, standing at 2.8. The model predicts that Preferences ${≻}_{8}$ will be those displayed by a decision maker with belief p.

Now the decision maker encounters the belief q, for example, she speaks with someone who reveals belief q, or she observes someone who displays belief q through the way she acts, or she gets other information which lets an alternative belief q emerge. Under q, the probabilities of ${≻}_{1},{≻}_{2}$, and ${≻}_{3}$ are 0.17, 0.15, and 0.08, respectively, and under q, it would be optimal to display preferences ${≻}_{4}$. Our decision maker assigns reliability of λ=0.3 to the new belief q, which means she has confidence that her current belief is correct at level 1−λ=0.7. Forming the corresponding synthesis belief $s_{0.3}$, she obtains the probabilities 0.1, 0.05, and 0.1 for the preference relations ${≻}_{1},{≻}_{2}$, and ${≻}_{3}$, respectively. The preference she will adopt under the synthesis belief is ${≻}_{6}$.

In all three instances, the preferences adopted under the synthesis belief are, according to the metric d(·,·), strictly “between” the preferences displayed under the beliefs p and q, meaning that the decision maker moves strictly closer toward the preferences she observes. However, this is not generally the case, and it is easy to find distributions p and q for the given values of λ where the choice based on the synthesis distribution is the same as the choice based on either p or q.

## 4 Experiment Design and Empirical Hypotheses

To test the model, we designed an experiment based on the seminal work of Holt and Laury (2002) that was already described at the end of Section 2. However, in order to test the predictions of Section 3, we added three additional stages.

The experiment was conducted at the *Centre for Research in the Behavioural Sciences* (CRIBS) at *Nottingham University Business School*. We recruited 248 students from various disciplines using ORSEE (Greiner 2015). Participants had no prior experience with a Holt and Laury (2002) experiment and were restricted to participating only once. Each participant received a £3 show‐up fee and an average of £3 for a 30‐min experiment. Four participants did not complete the survey, so we excluded their results from the dataset, resulting in 244 observations.

The experiment had three stages. In each stage, we used a modified version of the Holt and Laury (2002) lottery menu to measure individuals' attitudes toward risk (see Table 3 and the description of the Holt and Laury (2002) setup at the end of Section 2). Participants had to choose one of the options in the lower part of Table 3, which revealed the switching point where they wanted to switch from “Asset A” to “Asset B.” As can be seen in Table 1, the higher the switching point, the more risk‐averse is the participant.

Before the experiment started, participants received instructions about the experiment and their payoffs. We explained the three stages of the experiment, the decisions that participants would need to make in each stage, and how payoffs would be calculated. The payoff calculation proceeded as follows. One scenario would be selected randomly from any of the three rounds, and a fair die would determine the final payoffs of the selected decision (see Appendix B). This procedure was designed to prevent participants from attempting to play strategically. The experiment ran in z‐tree (Fischbacher 2007) and was followed by a short questionnaire. We will now describe the three stages of the experiment in more detail.

**Stage 1: Personal risk preference (we will refer to this stage as** ***Base*** **)** This stage was designed to determine participants' initial risk preferences. Participants were presented with the lottery menu of Holt and Laury (2002) (in the version shown in Table 3) and asked to decide their switching point by choosing one of the switching points from 0 to 10.^20^ The selected switching point indicates individual risk preferences: switching points from 0 to 3 imply risk‐seeking behavior because the decision maker prefers lotteries with bigger spread and lower expected value to lotteries with lower spread and higher expected value.^21^ Conversely, switching points from 5 to 10 are consistent with risk aversion. A risk‐neutral decision maker would therefore choose switching point 4, though subjects whose attitude is not exactly risk‐neutral but sufficiently close to risk‐neutral would choose switching point 4, too. Participants did not have a hard time constraint for making their decisions, and all participants made their decisions in a reasonable time frame so that there was no necessity to enforce a time limit.

Before stages 2 and 3, participants were given information about the switching points of a reference group as follows: “The average switching points of some participants in previous sessions was x.” The signal was predetermined to be fuzzy, that is, it was left open for participants to guess *how many* participants chose that switching point and which kind of reference group we were referring to. We set x to be 3 before stage 2 and 7 or 9 before stage 3. In this way, we avoided *deception*, as there had been a test run of the experiment where participants had chosen switching points 3, 7, and 9. When deciding on the signals that were communicated as signals to participants before stages 2 and 3, we centered around the most commonly found switching point of 5 (approximately, see Table 4 below) and then moved equidistantly into both directions. A share of 25% of students got signal nine. While a switching point of 7 lies just within the range of switching points found in the literature, the switching points of 3 and 9 lie clearly outside that range (see Table 4). A switching point of 3 clearly indicates that participants in a previous session were risk‐loving, while a switching point of nine suggests abnormally high risk aversion.

**TABLE 4:** Average switching points in the literature.

Source	Value
He et al. (2012)	4.48 (5.25, 4.84)
Bellemare and Shearer (2010)	4.62
Anderson and Mellor (2008)	4.98
Dave et al. (2010)	5.00
Holt and Laury (2002)	5.17
Harrison et al. (2005); Goeree et al. (2003)	5.3
Lusk and Coble (2005)	5.42
Baker et al. (2008)	5.67
Anderson and Mellor (2009)	5.68
Holt and Laury (2005)	6.10
Masclet et al. (2009)	6.6 (6.9)

Table 4 provides an overview of switching points found in the literature. The lowest switching point is reported by He et al. (2012). They report switching points for males (4.48), females (5.25), and couples (4.84) separately.^22^ The highest value of 6.6 was found by Masclet et al. (2009) who study individual attitudes toward risk. Moreover, they report a risk attitude of 6.9 for decision‐making in groups, suggesting that groups are slightly more risk‐averse than individuals and contradicting the so‐called *risky shift* phenomenon (Wallach et al. 1962; Aronson et al. 2017, p. 308). Most papers find a switching point of around 5, suggesting that the average decision maker is slightly risk‐averse.

While switching points are commonly interpreted as measures of individual risk aversion, they can also be understood as decision thresholds analogous to those encountered in high‐stakes HS&E contexts. In such settings, decision makers frequently face binary or discrete choices—such as whether to shut down a process, adopt a safety margin, or escalate a warning—under substantial uncertainty. The switching point thus captures the point at which a decision maker is willing to trade expected performance for increased safety, making it a natural proxy for precautionary behavior in HS&E risk management.

**Stage 2: Risk‐loving reference (we will refer to this stage as H3)** Before stage 2, participants were told that the average switching point in a selected session was three and then asked to choose once more their switching point from the same design that they were given in stage 1.

From an HS&E perspective, the information provided in stage 2 mirrors situations in which decision makers receive benchmarks or summaries of peer behavior, such as industry safety averages, best‐practice reports, or expert committee recommendations. These signals often shape subsequent safety‐related decisions, even when they are abstracted from the specific operational environment. Stage 2 therefore captures how exposure to comparatively risk‐tolerant reference behavior may influence the revision of safety thresholds and precautionary standards.

**Stage 3: Risk‐averse reference (we will refer to this stage as H7/H9)** In this stage, we split participants into two groups. The first group, 184 participants (75%), was told that the average switching point of a selected previous session was seven (H7), and the second group, 60 participants (25%), was informed that the switching point of a selected previous session was nine (H9). After receiving the information, participants were again asked to choose their switching point from the same setup that they were given in stage 1.

Stage 3 corresponds to a contrasting but equally prevalent HS&E scenario: the reception of highly precautionary reference information, such as reports following accidents, regulatory tightening, or evidence of near misses in comparable organizations. By varying the degree of risk aversion conveyed by the reference group, we are able to study whether decision makers respond proportionally to increasingly conservative signals, a key issue in understanding how safety standards escalate, or fail to escalate after adverse events.

**Stage 4: Payoff preference**. After the experiment, participants were queried about their preference regarding which of the three stages should determine their payoff. Given that participants understood the selection of the stage for calculating their payoff was random and their response would not influence their payout, their choice provides insight into their risk attitude after completing all three stages. This gives us an opportunity to separate the fleeting effects of information on their risk attitude from the more permanent ones.

In HS&E settings, organizations must often decide whether to adhere to initial safety assessments or adopt revised standards in light of new information. Stage 4 mirrors this trade‐off by allowing participants to select which of their previously adopted preferences should determine their payoff. This choice provides insight into whether decision makers regard updated preferences as superior and more reliable, or whether they prefer to rely on their initial judgment. As such, this stage captures an essential dimension of risk governance: the balance between stability of rules and adaptability to new evidence.

After participants finished the experiment, they were asked to fill out a questionnaire asking for their demographic characteristics, such as age, nationality, major, gender, and level of trust (Glaeser et al. 2000). The questions concerning social trust were trust in traffic controllers, foreigners (both domestic, that is, “strangers,” and international), accountants, and pilots. Regarding institutional trust, we enquired trust levels toward the police, public authorities, banks, and churches. A four‐level Likert scale was employed to measure the level of trust, ranging from “No trust at all” to “Little trust,” “Quite a bit of trust,” and “A lot of trust.” We utilize the factor analysis approach to construct the social trust index and institutional trust index.

Following Chmura et al. (2022), who found that finance students are less likely to follow leaders, we measured confidence by conducting an experiment with a separate group of finance students. In addition, we assessed social trust using a well‐established survey introduced by (Brehm and Rahn 1997; Fehr et al. 2002; Zmerli and Newton 2008). For institutional trust, we adopted Gallup's approach to measure trust levels in various institutions, such as the government, large corporations, the press, courts, and banks. To construct social trust and institutional trust indices, we employed the principal component analysis (PCA).

We will now state the theoretical predictions of the experimental results.

According to the model laid out in Section 2, if we confront somebody with a choice between lotteries, the decision maker aims at gauging the evolutionarily optimal level of risk aversion, and then she adopts the preferences associated with that level. In our experiment, this would be happening in the first stage. Accordingly, we interpret the preferences she displays in the first round as those associated with the level of r, she initially considers evolutionarily optimal for the choice problem at hand.

In the experiment, once the participant displays her preferences, we inform her about the average switching point of a reference group (the *signal*), and she is asked to choose again. Proposition 2 predicts that if her original choice corresponded to Interval j∈{1,…,n}, and she is informed that the average choice of the reference group corresponded to Interval k∈{1,…,n}, her new choice will correspond to an Interval weakly between j and k (i.e., j and k included). In terms of the associated switching points, this translates into:

Hypothesis 1 The switching point of the choice *after* information was obtained will be (weakly) between her original switching point and the switching point of the reference group.

In the model, the parameter λ captures the confidence in her a priori judgment relative to how much she trusts the choices of others. She may be very confident in her own assessment of the situation and mostly dismisses the information about others' choices, in which case her λ is close to 0; or she is rather insecure and assigns a lot of significance to what others do, in which case her λ is close to 1. The simulation (Table 2) suggests that the strength of the movement in response to the signal positively correlates with the value of λ.

The parameter λ can be interpreted having two components and is, in a conceptual sense, their *ratio*: (1) trust in other agents' judgments and (2) confidence in her own assessment.^23^ These two components can be proxied by traits of the decision makers; for example, “trust in authorities” may be a proxy for (1), and experience with risky choice and expertise in statistics may be a proxy for (2). We therefore state:

Hypothesis 2.1 Confidence in her own judgment is negatively correlated with how far the decision maker moves toward the signal.

Hypothesis 2.2 Trust in others is positively correlated with how far the decision maker moves toward the signal.

In the context of HS&E risk analysis, the parameter λ can be interpreted as capturing a fundamental tension between expert confidence and institutional trust. High confidence may reflect expertise and experience, but it may also lead to insufficient responsiveness to external warnings. Conversely, high trust in others can facilitate organizational learning but may also result in excessive conformity. By linking λ to measurable traits such as trust in institutions and confidence in judgment, our experiment provides a framework for diagnosing when social learning improves safety decisions and when it may undermine them.

In the experiment, subjects were given information about the average choices of reference groups *twice*. In the end of the experiment, they were asked to choose from which of the three preferences they adopted they want to receive their payoffs.

Our model suggests that from an evolutionary perspective, the adopted preferences can only get “evolutionarily better,” if anything, when additional information comes in. Information can never lead to a worse estimate: if the decision maker believed that the information is worthless because, say, the reference group has less understanding of the situation than herself, this would correspond to λ=0, and the information would have no impact on the preferences displayed. This leads to:

Hypothesis 3 Subjects want to have the payoffs from the round where they displayed their *most updated* preferences.

## 5 Experimental Results

Tables 5–and 7 present the individual risk preferences observed across the three treatments: Base, H3, and H7/H9. Table 5 presents the results for all participants (244), Table 6 for participants without finance backgrounds (184), and Table 7 for participants with finance backgrounds (60). On average, participants exhibit a risk‐averse mentality, with an average switching point of 6.475, which aligns with existing literature (e.g., Holt and Laury 2005; Masclet et al. 2009). The *p*‐values in the tables correspond to the null hypothesis that risk preferences in the Base and H3; H3 and H7/H9 are equal, using the Mann–Whitney U test.

**TABLE 5:** Individual risk preferences in the three stages, based on full sample.

	Base	H3	H7	H9
Switching point	6.475	6.209	6.793	6.466
*p*‐value (Base vs. H3)	0.001***			
*p*‐value (H3 vs. H7)	0.000***			
*p*‐value (H3 vs. H9)	0.063*			
*N*	244	244	184	60

*** *p* < 0.01, ** *p* < 0.05, * *p* < 0.1

**TABLE 6:** Individual risk preferences in the three stages, based on subsample of participants with no finance background.

	Base	H3	H7	H9
Switching point	6.666	6.282	6.790	7.090
*p*‐value (Base vs. H3)	0.000***			
*p*‐value (H3 vs. H7)	0.000***			
*p*‐value (H3 vs. H9)	0.046**			
*N*	195	195	162	33

*** *p* < 0.01, ** *p* < 0.05, * *p* < 0.1

**TABLE 7:** Individual risk preferences in the three stages, based on subsample of participants with finance background.

	Base	H3	H7	H9
Switching point	5.714	5.918	6.818	5.703
*p*‐value (Base vs. H3)	0.945			
*p*‐value (H3 vs. H7)	0.455			
*p*‐value (H3 vs. H9)	0.543			
*N*	49	49	22	27

In order to measure the strength of changing risk attitudes, we employ two binary variables which we call weak herding (*h‐weak*) and strong herding (*h‐strong*). *h‐weak* is 1 if a participant does not shift her risk preferences in response to the signal or moves toward the signal, and 0 otherwise. *h‐strong* assumes the value 1 only if a participant strictly changes her preference toward the signal (i.e., shifting toward 3 in stage 2 and toward 7 or 9 in stage 3), and 0 otherwise.

We define further binary variables to capture other dimensions of preference changes that allow for various interpretations and follow‐up analyses. These are magnitude of risk shifting (herding magnitude, *h‐mag*), direction of risk shifting (herding in both stages, *herd*), moving against the signals in both stages (*contrarian*), and the frequency of risk shifting (no strong herding once or twice, *H012‐strong*; no weak herding, once or twice *H012‐weak*). While we do not use these variables to test the hypotheses, they will serve to formulate some exploratory findings at the end of this section.

Table 8 presents the two main measures, and Table 9 exhibits the secondary measures.

**TABLE 8:** Preference changes among participants with and without financial training—primary variables.

	All	Nonfinance	Finance	*p*‐values
*h‐weak*	70.90%	73.85%	59.18%	0.043**
*h‐strong*	22.54%	24.62%	14.29%	0.122
*N*	244	195	49	

*** *p* < 0.01, ** *p* < 0.05, * *p* < 0.1

**TABLE 9:** Preference changes among participants with and without financial training—secondary variables.

	All	Non‐finance	Finance	p‐values
H Mag	0.803	0.953	0.204	0.037**
Herd (both)	26.23%	27.18%	22.45%	0.501
Contrarian (both)	4.92%	4.10%	8.16%	0.240
H012 weak	1.647	1.697	1.448	0.022**
‐ H0 weak	6.15%	4.10%	14.29%	
‐ H1 weak	22.95%	22.05%	26.53%	
‐ H2 weak	70.90%	73.85%	59.18%	
H012 strong	0.790	0.835	0.612	0.078*
‐ H0 strong	43.44%	41.03%	53.06%	
‐ H1 strong	34.02%	34.36%	32.65%	
‐ H2 strong	22.54%	24.62%	14.29%	
*N*	244	195	49	

*** *p* < 0.01, ** *p* < 0.05, * *p* < 0.1

**Original switching point and reference's switching point**

**FIGURE 1:**
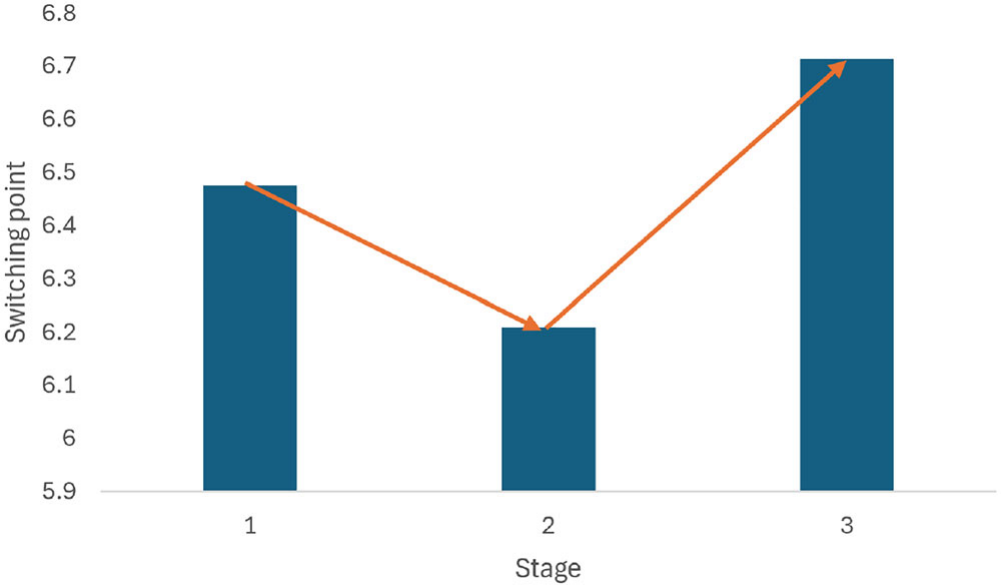
Risk preferences in the three stages followed the signals.

The findings reported in Tables 5–7 suggest that in treatments H3 and H7/H9, participants adjust their risk preferences in response to the provided signals, and these differences are statistically significant. This supports Hypotheses 1. More specifically, the average switching point in the full sample in treatment H3 was 6.209, while in H7/H9, it was 6.793/6.466 (Table 5). Figure 1 shows how the average switching point chosen by the participants follows the signals.

**Confidence, trust and risk preferences**

Results in Table 8 that show that 70.90% of participants exhibit weak herding behavior, while 22.54% engaged in strong herding are also supportive of Hypothesis 1.

The findings reported Tables 6 and 7 suggest that the decision to shift risk preferences toward the signals was statistically significant among nonfinance participants but not among those with finance backgrounds. While the switching point of nonfinance individuals is 6.666 in the Base, 6.282 in H3, and 6.790/7.090 in H7/H9, these numbers are 5.714, 5.918, and 6.818/5.703, respectively, for individuals with finance training. This finding is in line with Hypothesis 2.1, that is, individual characteristics such as financial training increase a participant's confidence in her own judgment. This is also consistent with results in Table 8 that participants without finance backgrounds are more likely to conform to informed risk preferences. Table 9 reveals similar results across different measures. In terms of the magnitude of risk‐shifting, h‐mag is 0.803 with all participants but is significantly higher with nonfinance participants. Overall, our findings support Hypothesis 2.1.

Next, we explore individual trust and its relationship with preference‐changing behavior. Specifically, we test if trust in others leads individuals to adjust their risk preferences stronger toward the signal. To explore this, we construct a social trust index and institutional trust index with PCA, based on various questions in the survey regarding both social trust and trust in institutions (Glaeser et al. 2000).

The results in Table 10 indicate that the level of trust has a negative effect on risk‐shifting behavior. Participants are less inclined to alter their risk preferences if they exhibit a high level of social and institutional trust (Table 11 B). However, the picture is not clear if one looks at individual questions. The correlations between *h‐weak* and “trust in courts,” “trust in the EU,” and “trust in foreigners” are 0.23, 0.15, and 0.22, all of which are significant with p<0.05.

**TABLE 10:** Adjustment of risk preferences, trust, and personality.

	Main risk‐shifting measures	
	h‐weak	h‐strong
Age	−0.108*	−0.037
Maths	0.125*	0.017
Risk preferences	0.059	−0.019
Social trust	0.080	−0.059
Institutional trust	0.008	−0.084
Extraversion	−0.041	−0.009
Agreeableness	−0.048	−0.045
Conscientiousness	0.050	0.046
Neuroticism	0.002	0.068
Openness	−0.027	−0.107*
*N*	244	244

*** *p* < 0.01, ** *p* < 0.05, * *p* < 0.1

**TABLE 11:** Individual characteristics and risk preference shifting.

	Other risk‐shifting measures				
	h‐mag	Herd	Contrarian	H 012‐weak	H 012 ‐strong
Age	−0.113*	−0.043	0.115*	−0.067	−0.116*
Maths	0.098	0.004	−0.061	0.133**	0.030
Risk preferences	0.085	−0.076	−0.166***	0.092	0.032
Social trust	−0.039	−0.106*	−0.054	0.083	−0.061
Institutional trust	−0.077	−0.127**	−0.060	0.020	−0.100
Extraversion	0.014	−0.009	−0.068	−0.028	0.039
Agreeableness	−0.021	−0.002	−0.009	−0.046	0.002
Conscientiousness	0.139**	0.015	0.051	0.048	0.124*
Neuroticism	−0.000	0.052	0.059	−0.005	−0.011
Openness	0.006	−0.105	0.029	−0.031	−0.020
*N*	244	244	244	244	244

*** *p* < 0.01, ** *p* < 0.05, * *p* < 0.1

Taken all of this together, we *weakly reject* Hypothesis 2.2.

**Updated risk preferences**

After the first three stages, participants were asked from which stage they would prefer to receive their payment. Overall, 29.10% of participants opted for payment based on their original decisions in the first stage, while 38.11% preferred payment based on the second stage results, and 32.79% preferred payment from the third stage. For testing Hypothesis 3, we excluded participants who made the same choice in all three stages.

For those who remain, the question is whether participants tended to choose the payoff from the last stage in which they shifted their risk preferences. The first two columns of Table 12 show that 50% of participants who shifted in stage 2 and remained the same in stage 3 selected the payoff from stage 2 (in fact, 83.33% chose the payoff from the last stage in which they shifted, since in this case, risk preferences in stages 2 and 3 were the same). Additionally, 43.10% of participants who shifted in both stage 2 and stage 3 selected the payoff from stage 3. This is consistent with Hypothesis 3. Results in the last column are not consistent with Hypothesis 3, only 26.92% of participants who remained the same in stage 2 and shifted in stage 3 selected the payoff from stage 3. However, it appears that the behavior of majority of participants is consistent with Hypothesis 3. It should also be noted that the behavior of those participants who did not change their preferences at all, whom we did not include in Table 12, is in line with Hypothesis 3. It is also worth pointing out that there was a relatively small sample size of participants who changed their preferences twice, namely, only 52 participants.

**TABLE 12:** Payoff preferences among participants who shifted their risk preferences.

	Shift in S2 & stay same in S3	Shift in S2 & S3	Stay same in S2 & shift in S3
Payoff 1	16.67%	26.72%	23.08%
Payoff 2	**50.00%**	30.17%	50.00%
Payoff 3	**33.33%**	**43.10%**	26.92%
*N*	24	116	52

*** *p* < 0.01, ** *p* < 0.05, * *p* < 0.1

We also found some noteworthy results not directly related to the model. First of all, we examined whether gender influences the decisions to shift individual risk preferences. The results in Table 13 indicate that females are more inclined to shift their risk preferences. Specifically, 26.87% of females exhibit a strong shift, compared to only 17.27% of males, and this difference is statistically significant. Similarly, 30.60% of females follow the signals (herd) in both stages, while this percentage is 20.91% for males. Additionally, the frequency of strong risk shifting is significantly higher among females; 26.87% of females make strong shifts twice, compared to 17.27% of males. There is a body of literature on herd behavior and gender. For instance, studies such as Gupta and Goyal (2024) and Talpsepp and Tänav (2021) find no gender gap in following influencers, while Zheng et al. (2021) suggest that female investors are more likely to herd in the Chinese stock markets. Our result contributes to this literature by highlighting the gender gap in individual risk preference shifting.

**TABLE 13:** Changing risk preferences and gender.

	All	Female	Male	*p*‐values
*H* (weak)	70.90%	72.39%	69.09%	0.573
*H* (strong)	22.54%	26.87%	17.27%	0.075*
*N*	244	134	110	

*** *p* < 0.01, ** *p* < 0.05, * *p* < 0.1

Table 14 presents other correlations between individual characteristics and the change of risk preferences. The results show that age is negatively and significantly correlated with h(weak), hmag, and h012weak, while positively and significantly correlated with contrarian behavior. As participants grow older, they are less likely to shift their risk preferences toward the signals but against the signals. This finding supplements the literature regarding age and herd behavior. Lamont (2002) indicates that age is negatively correlated with hearing decisions among American forecasters, while Ashiya and Doi (2001) claim that Japanese forecasters herd regardless of their age.

**TABLE 14:** Individual characteristics and risk preference shifting.

	H (weak)	H (strong)
Age	−0.108*	−0.037
Maths	0.125*	0.017
Risk preferences	0.059	−0.019
Social trust	0.080	−0.059
Institutional trust	0.008	−0.084
Extraversion	−0.041	−0.009
Agreeableness	−0.048	−0.045
Conscientiousness	0.050	0.046
Neuroticism	0.002	0.068
Openness	−0.027	−0.107*
*N*	244	244

*** *p* < 0.01, ** *p* < 0.05, * *p* < 0.1

The Big Five personality traits also have significant impacts on risk preference shifting, wherein conscientious participants are more likely to conform to the informed risk preferences with a greater magnitude, while open participants tend to make the opposite decisions.

## 6 Discussion

We interpret the experimentally elicited switching points as *risk‐acceptability thresholds*, analogous to the safety cutoffs that guide decisions in HS&E contexts. From this perspective, changes in switching points across stages reflect the updating of safety thresholds in response to social and benchmark information, a central mechanism in adaptive risk management. The experimental results therefore shed light on why decision makers respond heterogeneously to identical risk signals, with some exhibiting inertia and others substantial adjustment.

Analyzing the initial switching points in the Base treatment reveals that participants with financial training are more likely to take risks compared to nonfinance participants (5.714 vs. 6.666, *p*‐value = 0.048). This suggests that before any information exchange, confidence in one's own judgment, that is, the inverse value of λ, is negatively correlated with risk aversion. This may reconcile our findings with the known phenomenon of *risky shift* (Wallach et al. 1962), which is the observation that group decisions tend to be less risk averse than individual decisions. Assuming that group decisions are based on information sharing among the group members, the “group λ” will usually be lower than the individual λʻs of the group members so that the group acts more risk‐loving than the individual members.

Interpreted through an HS&E lens, the initial switching point can be read as a safety threshold—a cutoff at which a decision maker is willing to sacrifice expected performance (or convenience) for reduced downside risk. In many HS&E settings, analogous cutoffs determine whether a process continues, whether precautionary measures are adopted, or whether activities are halted under uncertainty. The observed difference between finance and nonfinance participants therefore suggests that domain training may shift the location of such thresholds even before any social information is introduced, with implications for heterogeneity in precautionary decision‐making within teams and across roles (e.g., operators, engineers, and managers).

A question is why Hypothesis 2.2 was rejected. One possibility would be that the model parameter λ is determined only by confidence in one's own judgment, as suggested by the confirmation of Hypothesis 2.1, but not by trust in others. This would not challenge the model itself but rather our ex‐ante interpretation of the λ parameter, not requiring a change to the architecture of our theory.

From the perspective of HS&E risk analysis, the rejection of Hypothesis 2.2 may reflect that the relevant construct is not “trust in others” in a general, survey‐based sense, but rather credibility‐weighting of specific information channels (e.g., trust in regulators, incident databases, safety leadership, or technical experts) (Cox et al. 2006). In operational environments, decision makers often exhibit high trust in some sources (e.g., internal engineers) and low trust in others (e.g., corporate messaging), and this source‐specific credibility is precisely what governs whether safety thresholds are updated. Thus, our null finding may indicate a measurement mismatch: the “trust” items capture a broad disposition, while the model's λ may be driven by contextual trust in the relevance and competence of the reference group.

There are also more general objections against the idea that people are optimizing an evolutionary parameter through the adoption of preferences. One is the apparent lack of introspective support. We, and most people with whom we discussed our theory, do not perceive decision situations, not least choices between *lotteries*, to be about maximizing offspring and survival. Introspectively, many people question whether they are investigating their preferences *at all* when choosing among lotteries. Rather, they think that they base their decisions on quantitative aspects of those lotteries, like their expected values. This cognitive understanding is built into our theory, where experience and cognitive understanding influence the parameter λ, but for many people, the cognitive component seems not to square with the idea that preferences result form an underlying evolutionary optimization.

Our response to these objections is that if the processes posited in this paper happen, then they must be subconscious, at least when it comes to their hypothesized evolutionary foundations. A person choosing among lotteries will not ask: “What risk should I take to optimise my evolutionary outcome?” Rather, she will ask: “What risk *feels right* for this situation?”

This mechanism is particularly plausible in HS&E contexts because many safety decisions are made under time pressure, ambiguity, and incomplete probabilistic knowledge (Pooladvand and Hasanzadeh 2022; Pawar and Velaga 2022). Even when formal risk matrices or quantitative risk assessments are available, day‐to‐day choices frequently rely on intuitive thresholding—whether a situation “looks unsafe,” whether conditions “seem within limits,” or whether a deviation “feels acceptable.” Our framework provides a disciplined way to interpret such judgments as the output of preference formation and updating rather than as noise or purely cognitive calculation.

This does not mean, however, that her cognitive apparatus, her *understanding of the situation*, has no influence on her preferences. To the contrary, like many other preferences and emotions, risk preferences are almost surely *cognitively moderated* (see, e.g. Zajonc and Markus 1982; Schkade and Johnson 1989; Betto 2024). To illustrate what this means, consider another evolutionarily consequential decision the reader is certainly familiar with, namely, whether or not to eat another portion of powdered‐sugar doughnuts.^24^ If you have to make this decision, you will primarily interrogate your appetite and hunger and not consciously analyze the effects of the doughnut consumption on your reproductive success. Nonetheless, your desire for the doughnut may be *cognitively moderated*: awareness of the facts that eating the doughnuts may impact your body weight or blood sugar levels may make you resist the temptation, override the immediate craving for sugar, and possibly impact your preferences to an extent that you do not *want* to eat the doughnuts anymore (White et al. 2020; Calabro et al. 2023, survey research on the impact of cognitive processes on food preferences).^25^ On the other hand, if you know that a 30‐mile hike is ahead of you, you may have no qualms whatsoever to eat the doughnuts, and you may actually *enjoy* them more than if you were mostly concerned with your weight. In both cases, conscious considerations feed back to the basic preference level.

In HS&E practice, this type of cognitive moderation corresponds to the effect of training, procedures, and decision aids: technical knowledge can shift which risks are attended to, how salient worst‐case outcomes feel, and how strongly new information is weighted. In our model, this maps naturally into λ as the relative weight on one's own assessment versus socially transmitted information. The empirical differences by financial training are therefore consistent with the broader HS&E premise that expertise can stabilize thresholds, sometimes appropriately (robust judgment), sometimes inappropriately (excessive confidence).

It was first pointed out by Maslow (1943) that some preferences are highly instinctual, while others are more intellectual; along this spectrum, it seems likely that preferences for lotteries belong to the latter.^26^ However, as the above examples show, even if sophisticated cognitive reasoning is involved in a decision‐making process, the outcomes may still be genuine *preferences*, that is, *valence* attached to different options, in the same way as the desire for vengeance can be felt viscerally even though it may depend on a sophisticated cognitive interpretation of a complex social situation.^27^

Cognitively moderated preferences can, like all other preferences, be grounded in evolutionary history. The optimal risk attitude when deciding whether or not to attack a dangerous but nutritious animal, like a mammoth, will depend on various parameters: How large and dangerous is the animal, and how likely are different outcomes that can result (e.g., the mammoth being killed, oneself being killed or injured, or oneself having to abandon the attack)? How are these probabilities affected by the weapon that is available (e.g., spear, stone, club) and by the current physical fitness? How likely is it that other members of the horde will join the attack? And, depending on their contribution to the project, what share of the spoil will they claim if the attack was successful? The answers to these questions depend on cognitive analysis, but they lead to the emergence of *genuine preferences*, namely, the appetite for taking the risk of attacking the mammoth.

Another aspect of our theory not intuitive to everyone is the *context dependence* of the evolutionarily optimal level of risk aversion. However, context dependence is an obvious fact for many other preferences:^28^ the desire for food does not emerge if one has just eaten, a lust for aggression or wish to flee usually requires to be in a fighting or conflict situation, and social preferences, say, for harmony, may depend on who are the people one is interacting with.

The context dependence of risk preferences has been discussed for a long time (Wolf and Pohlman 1983), and there is considerable empirical affirmative evidence from different domains (e.g., Karni et al. 1983; Barseghyan et al. 2011; Einav et al. 2012; Schildberg‐Hörisch 2018). There is, of course, also the related debate about the connection between risk aversion and a decision maker's *wealth* (which can be considered a part of the *context*),^29^ although the empirical picture regarding the correlation between risk aversion and wealth is not unambiguous (see the findings and the discussion of the empirical literature in Rieger et al. 2015). Summing up, we do not think that context dependence is an aspect of our theory that is difficult to accept.

Importantly, the contribution of our experimental results to HS&E is not that a lottery task directly reproduces industrial or public‐health hazards, but that it isolates a general mechanism that those environments depend on how safety thresholds move when decision makers receive social information. Because HS&E risk governance frequently relies on benchmark comparisons, incident learning, and expert consensus signals, understanding when individuals update, how far, and which traits predict responsiveness, speaks directly to the design of effective risk communication and organizational learning processes.

## 7 Relevance in Health, Safety, and Environment Contexts

In public health contexts, two largely separate determinants of behavior in situations involving risk have been identified. The first one is, as may have been expected, the *risk attitude*. Experimentally measured risk aversion predicts health‐related behaviors such as smoking, heavy drinking, obesity, and seatbelt use (Anderson and Mellor 2008). Even subtle framing differences in risk‐elicitation tasks can shift measured preferences and change behaviors (Yang et al. 2022; Van der Pol and Ruggeri 2008).

The second determinant are *peer effects*, sometimes leading to “contagiousness” of harmful activities like smoking, alcohol, and drug consumption (Alexander et al. 2001; Fergusson et al. 2002; Duncan et al. 2005; Powell et al. 2005; Lundborg 2006; Clark and Lohéac 2007). In psychological research, however, peer effects are typically not explained as the result of information transmission and learning from others, as it is the case in our theory, but as a result of, for example, social norms (Tajfel et al. 1971), reciprocity (Vaquera and Kao 2008; Lin and Weinberg 2014), and group identity (e.g., Goette et al. 2006; Charness et al. 2007; Chen and Li 2009; Gioia 2017, the last paper focusing on risk‐taking behavior).^30^ Our evolutionary hypothesis combines and extends both explanations: the existing risk preferences matter, but peer effects, which work through information sharing, contribute to the formation of those preferences.

Our model and experimental results can also explain the dynamics of whether and when risk preferences change. Arguably, this aspect has at times been neglected as much of the existing literature views or finds (risk) preferences to be basically stable (Stigler and Becker 1977; Andersen et al. 2008; Dasgupta et al. 2017).^31^

In contrast, and in line with the ideas we advance in this paper, a substantial body of research in HS&E shows that risk preferences and risk acceptability can shift frequently, even in routine, noncrisis settings. In line with our theory, risk‐taking in these contexts is often shaped by local experiences, perceived exposure to hazards, and social cues and peer behavior. For example, views on pesticide policies in Canadian cities are not primarily driven by general environmental preferences but by residents' personal experiences with chemical use and conflict over the use of pesticides (Hirsch and Baxter 2011). In public health, shocks and framing effects can trigger short‐run shifts in risk attitudes (Decker and Schmitz 2016; Van der Pol and Ruggeri 2008), consistent with the idea that risk preferences are adjusted in response to new information.

Environmental valuation studies also demonstrate these dynamics: willingness to pay for mortality risk reductions varies with local pollution levels and the salience of specific causes of death (Lindhjem et al. 2011; Guignet and Alberini 2015). Likewise, workplace safety research demonstrates how information acquired through communicating with and observing others can alter perceived risks and protective behavior (Wakefield et al. 2010; Dean 2014; Maglio et al. 2016; Wong et al. 2020; Cakir et al. 2023).

Across these domains, HS&E research consistently shows that risk preferences and risk acceptability are dynamic and influenced by social learning, trust, and domain‐specific knowledge, precisely the mechanisms for which our framework provides an evolutionary foundation.

### 7.1 Escalation of Safety Thresholds Following Process Safety Deviations

A central challenge in occupational and industrial safety is deciding whether ongoing operations should be halted, modified, or allowed to continue following indications that safety margins have been eroded. In many high‐risk industries, such as chemical processing or refining, catastrophic accidents are often preceded not by a single dramatic event but by a series of process deviations, warning signs, or near‐miss incidents that signal elevated risk without immediately causing harm. Well‐documented cases, such as the events preceding the Texas City refinery explosion, illustrate how such warning signals can be interpreted very differently across organizations and over time (Reason 1998; Vaughan 1996; Phimister et al. 2003).

From an HS&E risk analysis perspective, these decisions can be understood as threshold problems: managers and engineers implicitly compare their perceived probability of catastrophic failure against an acceptable risk level that determines whether operations are stopped, modified, or continued. If perceived risk exceeds this threshold, shutdowns or corrective actions are triggered; if not, operations proceed (Kang 2024; Grechuk and Zabarankin 2014). This structure closely parallels the experimental switching point in our design, which captures the cutoff at which decision makers are willing to trade expected performance for reduced downside risk.

Information about safety deviations rarely comes in isolation. Organizations typically interpret local warning signs in light of external reference information, such as incident statistics from peer facilities, industry benchmarks, regulatory guidance, or past operational experience. In terms of our model, these reference points constitute a *signal* about the operationally appropriate level of risk tolerance in the given context. Our framework predicts that the extent to which safety thresholds are revised in response to such signals depends on the parameter λ, which captures the relative weight placed on external information versus confidence in local judgment. In work‐safety settings, λ can be interpreted as the balance between trust in industry‐wide safety knowledge and confidence in site‐specific expertise, engineering assessments, and operational familiarity.

Two distinct failure modes follow naturally from the model. When confidence in local judgment is very high (i.e., λ is low), organizations may insufficiently update their safety thresholds despite accumulating warning signals, leading to the normalization of deviance and the gradual acceptance of elevated risk. Conversely, when reliance on external benchmarks is strong and confidence is low (i.e., λ is high), organizations may overadjust to salient but noisy signals, triggering overly conservative shutdowns or costly operational disruptions.

From a risk management perspective, the framework highlights that improving work‐safety decisions is not only a matter of collecting more safety data, but also of understanding how different actors weight internal experience against external benchmarks. Effective HS&E governance therefore requires not only transparent reporting of safety signals, but also mechanisms that calibrate confidence appropriately, distinguishing between credible systemic warnings and transient noise, and tailoring communication to the expertise of decision makers.

### 7.2 Extensions to Other HS&E Domains

Similar threshold‐updating problems arise in public health decision‐making. Individuals and policymakers must decide whether to adopt, maintain, or relax preventive measures, such as vaccination, mask usage, or dietary guidelines, based on perceived health risks. These decisions depend on implicit acceptability thresholds for adverse outcomes, such as infection or morbidity risks. Typical signals include public health guidelines, expert consensus statements, epidemiological benchmarks, and observed compliance or behavior within peer groups (Morgano et al. 2022; Neumann et al. 2022; Meng et al. 2023). In terms of our model, these thresholds correspond to switching points, the external information constitutes the signal, and the parameter λ captures the balance between confidence in one's own assessment and trust in public health authorities or peer behavior. A key implication is that uniform risk communication may induce heterogeneous responses: highly confident individuals may underreact to credible guidance, while less confident individuals may overadjust to salient but noisy signals, suggesting a role for targeted and differentiated communication strategies.

Environmental risk management likewise involves the formation and revision of risk‐acceptability thresholds, for example, in setting permissible pollution levels, approving infrastructure projects, or determining acceptable failure probabilities for environmental hazards (Mourato 2006; Fei et al. 2023). Decision makers often rely on external reference points such as regulatory standards, international benchmarks, peer jurisdictions, or high‐profile environmental incidents. Within our framework, these reference points act as signals that inform updates of acceptable risk thresholds, with λ reflecting confidence in local expertise relative to trust in external standards and institutions. The model predicts that differences in this balance help explain why some authorities rapidly align with external benchmarks, while others resist adjustment. From a design perspective, the framework highlights the importance of calibrating benchmark information to institutional context in order to promote adaptive learning without triggering either inertia or excessive precaution.

## 8 Conclusion

This paper offers a decision‐theoretic model in which the adopted preferences depend on an underlying evolutionary parameter. Agents have different confidence in their ability to gauge that parameter and use the observation of other decision makers to improve their estimates. This allows for an experimental test of the model which we have carried out. By and large, the results are supportive of the model.

Interpreted through an HS&E lens, the model and experimental findings speak directly to how risk acceptability thresholds are formed and revised when individuals observe the behavior or judgments of others. In many HS&E domains, such as occupational safety, environmental regulation, and public health decision makers rely on benchmark information, incident statistics, expert panels, or peer practices to assess whether risks remain within acceptable bounds. Our results show that such information does not affect all decision makers uniformly: responsiveness depends systematically on confidence, experience, and the perceived value of others' judgments. This insight is central for understanding why safety standards sometimes update rapidly following new information and at other times remain surprisingly inert.

Our experiment also contributes empirical evidence on the questions of stability and influence factors of risk preferences. As future work, we may check whether the specific sequential setup of the experiment may have benefits for addressing some of the open question.

The framework developed in this paper directly contributes to HS&E research by offering a microfounded explanation for how context‐dependent, socially informed changes in risk acceptability arise, even in the absence of major external shocks. Many HS&E decisions, from health‐related behaviors and safety compliance to environmental risk perception and valuation, are known to be shaped by context, social learning, and the observation of others (see the literature surveyed in Section 7). Our model provides a structured mechanism for understanding how such context‐dependent and socially informed adjustments in risk attitudes can arise. Future work might apply our model of evolutionary driven preference dynamics to explain specific shifts in health‐risk behavior described in the literature (e.g., preventive actions or risky consumption) and workplace safety responses to leadership signals.

Beyond explaining observed behavior, our framework has practical implications for HS&E risk management. It suggests that the effectiveness of risk communication depends not only on the content of information but also on the confidence structure of the audience. Highly confident experts may appropriately discount weak signals but dangerously ignore credible warnings, while less confident decision makers may overreact to salient but uninformative cues. Recognizing this heterogeneity can inform the design of safety reporting systems, expert elicitation procedures, and organizational learning mechanisms aimed at improving decision quality under uncertainty.

In this way, our evolutionary theory could not only contribute to behavioral decision theory but also to the broader HS&E literature concerned with how individuals adapt their risk preferences across different environments and informational settings.

While HS&E contexts provide a particularly salient domain for applying our framework, given their reliance on thresholds, precaution, and learning from others, the underlying mechanism is more general. Beyond HS&E may be other applications of the model framework developed in this paper. As mentioned, the evolutionary parameter does not need to be *risk aversion* but could also represent degrees of other behavioral factors which affect the prospective number of offspring. Like in the case of risk aversion, a *thrive function* could map such underlying factors into actual, observable preferences.

For example, *patience* plays an important role in conflict situations. If someone demands a change of behavior from someone else, the latter should be given some time to change their ways before punitive measures are taken. Waiting not long enough may lead to an unnecessary escalation of the conflict with negative evolutionary consequences (including violence, injuries, and death). Yet, waiting too long with following‐up on a threat or demand that was made will lead to a loss of credibility, yielding disadvantages in future negotiations and conflicts. Like in the case of risk aversion, there is likely an *evolutionarily optimal* level of patience. Analogous trade‐offs arise in HS&E contexts, for example, in decisions about when to escalate safety concerns, enforce compliance, or intervene following early warning signs, where acting too quickly or too slowly can both have severe consequences.

Another example is *empathy*. Being empathetic toward nonrelatives can be evolutionarily advantageous because it may, among others, initiate evolutionarily beneficial tit‐for‐tat relations (see Axelrod and Hamilton 1981, and Chapter 12 in Dawkins 2016). However, when empathy is extended too generously, it can be exploited by others and then *reduce* the expected number of offspring. As before, this reasoning gives rise to the idea that there is an evolutionarily optimal level of empathy. In organizational safety settings, such dynamics may manifest in how managers respond to worker concerns, near‐miss reporting, or whistleblowing, where excessive skepticism can suppress valuable information while excessive empathy may undermine enforcement and accountability.

With these initial thoughts, we leave it to future research to explore the applicability of the model framework introduced here to these and other behavioral parameters, both within HS&E risk analysis and in broader decision environments where learning, trust, and context shape adaptive behavior under uncertainty.

## Endnotes

1 Game theorists have devised formal models to explain how the preference formation proceeds, for example, Samuelson (2001), Heifetz et al. (2007), Dekel et al. (2007). These models are based on the assumption that, on a fundamental level, genes are “players” (Dawkins 2016), underpinning the extensive application of game theory in theoretical biology (e.g., Alexander 2023; Lewontin 1961; Smith 1982; Weibull 1997).

2 This is not the first paper to theorize and speculate about the evolutionary origins of risk preferences. Similar ideas have been developed in different disciplines, notably in philosophy (e.g., Szpiro 1997; Okasha 2007; 2011), theoretical biology (Zhang et al. 2014; Heller and Nehama 2023), and (evolutionary) psychology (Barrett and Fiddick 2000; Slovic et al. 2004; Homma and Takezawa 2024).

3 A strict total relation ≻ is transitive, asymmetric (if a≻b, then not b≻a) and connected (if a≠b, then [a≻b or b≻a]). A strict total order does not allow for indifference between unequal elements, cutting away a great deal of case distinctions and other hassle without changing the model qualitatively.

4 We opt for restricting the range of r because in Section 3, we will model an agent's *belief* about the correct value of r as a density function. Total ignorance about r would correspond to a uniform distribution, which is not defined on an unbounded support. Moreover, note that r ranges in the *open* interval $\left(\underline{r},\bar{r}\right)=\left\{\;r\in R\;\;|\;\;\underline{r}<r<\bar{r}\;\right\}$, meaning that we require r to be an *interior value* of the closed interval $\left[\underline{r},\bar{r}\right]=\left\{\;r\in R\;\;|\;\;\underline{r}\leq r\leq \bar{r}\;\right\}$. In the subsequent analysis, this technical assumption helps us to avoid complications that arise from the special cases $r=\underline{r}$ and $r=\bar{r}$. From a practical perspective, the assumption is innocent because our analysis holds for arbitrarily large intervals $\left(\underline{r},\bar{r}\right)$. If in a specific application, the cases $r=\underline{r}$ and $r=\bar{r}$ are relevant, it is possible to work with a larger interval $\left({\underline{r}}^{*},{\bar{r}}^{*}\right)⊃\left[\underline{r},\bar{r}\right]$ for which $\underline{r}$ and $\bar{r}$ are interior values.

5 It is not easy to come up with a meaningful name for a function in a decision‐theoretic model. Initially, we used the term *preference function*, but that name could mean anything. The name thrive function reflects the idea that if an animal or human adopts the preferences selected by the thrive function, he or she will thrive in an evolutionary sense.

6 The reader may notice a similarity between the values defined in Lemma 1 and David Bell's (1988) concept of *one‐switch utility functions*. We are grateful to Peter Wakker for making us aware of this conceptual connection.

7 As we are working with strict relations, we could equivalently have written the condition in the set as $l^{\prime }{≻}^{\prime }l^{\prime \prime }\wedge l^{\prime }{⊁}^{\prime \prime }l^{\prime \prime }$.

8 If one considers a relation to be a subset of L×L and lines up the ${\left|L\right|}^{2}$ positions in L×L in a way that allows to express every relation as a sequence of zeros and ones (i.e., 0 if a pair is not in the relation and 1 if it is), the standard Hamming metric measuring the distance between those sequences would have double the value of the metric defined above applied to the original relations.

9 We assumed that the metric d(·,·) had already been used elsewhere but could not find an exact match. Closest may be the *Swaps Index* in Apesteguia and Ballester (2015), which measures how many pairwise comparisons have to be reversed in a preference relation R in order to make it consistent with an empirical observation. As the empirical observation is not a full preference relation but a single choice that is potentially inconsistent with R, their measure does not constitute a metric on the set of preference relations. Halevy et al. (2018) do define a measure of how close two rankings are. As their measure is based on a loss function, and since they are dealing with budget sets, it is not the same as the metric above. We are grateful to Yoram Halevy for these literature pointers.

10 The definition of linearity may at first sight look asymmetric, but it can be shown that if it is fulfilled, then there are also values $r^{3},r^{4}$ with $r^{\prime }\leq r^{3}<r^{4}\leq r^{\prime \prime }$ such that for all $r\in (r^{3},r^{4})$, it holds $d(r^{\prime },r)=1$.

11 Some experiments replicating Holt and Laury (2002) are listed in Table 4. Holt and Laury (2002) was recognized as the 5th most cited paper in the *American Economic Revue* published between the years 2000 and 2010 (Brice and Montesinos‐Yufa 2019). As of January 2025, Google Scholar lists a whopping 8532 citations of the paper.

12 All CRRA utility functions are positive affine transformations of the function

$$U\left(x\right)=\left\{\begin{array}{cc} x^{1-r} & \;\text{for}\;r<1\\ \ln x & \;\text{for}\;r=1\\ -x^{1-r} & \;\text{for}\;r>1, \end{array}\right.$$

for which the Arrow–Pratt measure of relative risk aversion equals r (for details, see Wakker 2008).

13 If we would use another function from the CRRA family, the r‐intervals associated with each switching point would change, but the preference relation associated with the switching point would be the same.

14 Without even using the specific form of the CRRA utility function, we can also recover most of the preference relation based on first‐order stochastic dominance and transitivity (assuming that there is only one switching point). In the above case, we know by first‐order stochastic dominance that $A_{10}≻A_{9}≻\text{…}≻A_{2}≻A_{1}$ and $B_{10}≻B_{9}≻\text{…}≻B_{2}≻B_{1}$. By transitivity then follows from switching point 5 not only that $A_{5}≻B_{5}$, but also $A_{5}≻B_{4}$, $A_{5}≻B_{3}$, and so on. Further such arguments are possible, but some comparisons remain undetermined if the utility function is not used to calculate the expected utility of the lotteries.

15 Our payoffs are not in dollars but in *Experimental Currency Units* (ECU), and the numerical values are 10 times larger.

16 This is guaranteed if the CRRA utility function generates a thrive function that is single‐crossing and linear.

17 If r is not risk aversion, the single‐crossing property requires a different motivation.

18 The possibility that the thrive function may be different for different decision makers may be relevant in some contexts, for example, for analyses outside of an evolutionary steady state, or if the Agents A and B come from separate populations that were exposed to different evolutionary pressures.

19 The existence of multiple medians is an unlikely case if the random variable derives from messy real‐world scenarios. Specifically, if the probability mass function ${\left\{p\left(i\right)\right\}}_{i=1,\text{…},n}$ is itself (partly) determined by random factors, and we can therefore consider it to be an n‐dimensional continuous random variable, then the probability of multiple medians is 0.

20 For a screenshot of the menu that was presented to the participants as well as the instructions given to the subjects, see Appendix B.

21 From here, one can derive that the decision maker has the risk attitude of risk‐seeking in its standard definition, that is, she prefers a lottery over having its expected value as a sure payment.

22 Their finding confirms previous results according to which women typically hold less risky assets compared to men, for example, Barsky et al. (1997), Jianakoplos and Bernasek (1998), Sunden and Surette (1998) (though Schubert et al. 1999 is not in line).

23 A parameter like λ may also drive the inclination of students to copy other students' answers in an assessment situation: the more confidence one has in one's own competence, and the less one trusts others, the lower will be the tendency to peek at the exam scripts of other students (Franklyn‐Stokes and Newstead 1995; Khodabakhshzadehs and Shoahosseini 2021).

24 Some reader may note that the craving for powdered‐sugar doughnuts features prominently in the book *Why Buddhism is True* (Wright 2017), which argues that Buddhism is a true religion in the sense that it anticipates central insights from evolutionary psychology.

25 In most decision situations, the cognitive apparatus is balancing and juggling multiple desires, which can be seen as independent behavioral drives as in the evolutionary psychology version of the *modular theory of mind* (Kurzban 2011). In this context, the rejection of the powdered‐sugar doughnut can be interpreted as the overriding of your immediate craving by a more fundamental survival or mating instinct (say, because of the increased risk of heart disease and because of the negative impact of overweight on mating market outcomes), instincts that are likely to be cognitively moderated themselves.

26 The need for *sleep*, on the other hand, is highly instinctual, as shown by the fact that it is difficult to override fatigue even if the cognitive apparatus recognizes that in a given situation, the pursuit of this desire is detrimental (there is a body of literature on the interplay between cognition and sleep, for example, Tousignant et al. 2019; Ballot et al. 2021; Cárdenas‐Egúsquiza and Berntsen 2022). This is illustrated by the fact that people struggle to resist fatigue even when that is extremely dangerous (e.g., while driving), despite an awareness of the danger and a determination to stay awake (Williamson et al. 2011). At the other end of the spectrum are various preferences that are highly contingent on a cognitive understanding of a situation. For example, genuine emotions of aggression and vengeance, and the preferences derived from those feelings, may depend on a cognitive interpretation of a situation, for example, these emotions may follow if one was insulted publicly but may be absent if the altercation happened in a private setting (Kim et al. 1998; Ijzerman et al. 2007; Torres and Bergner 2010). This difference may result from the potential status loss following from a meek response, which only enters the cognitive calculus in the public case (Santos et al. 2011). Similarly, retaliatory desires only emerge in the presence of a human adversary, not if the offender is a computer (De Melo and Gratch 2015; Adam et al. 2018), a distinction that requires strong cognitive input as it can only be made by a mind who holds a notion of the philosophically nontrivial difference between a person and a machine (Chalmers 1997). The idea that preferences are not only cognitively moderated but even serve *strategic* goals is at the heart of the game‐theoretic solution concept of *mental equilibrium* (Winter et al. 2017).

27 Lotteries as choice objects may appear to be dull, but this is not always the case: *excitement* is one of the main motivating factors for *gambling* (Lee et al. 2007), which, on an abstract level, is usually not much more than choosing between lotteries.

28 This was pointed out both in theoretical and empirical papers, for example, Ciampi et al. (1982), Karni et al. (1983), Mazar et al. (2014).

29 In expected utility theory, risk aversion follows from the degree of concaveness of a utility function. If that function is “more concave,” then the risk premium for a given lottery and the Arrow–Pratt measure associated with that utility function have higher values (Arrow 1974). Because a strictly concave utility function of money that is strictly increasing inevitably converges to a linear function (to be precise: a straight horizontal line) when the argument of the function approaches infinity, it follows that for a given lottery, the decision maker who maximizes expected utility will adopt a risk attitude that more and more resembles risk neutrality the higher is the wealth level affected by the lottery outcomes. This point can be illustrated with the following sketch: **art.eps**

30 Information sharing and “advice” was identified as one source of peer effects in Lomi et al. (2011), and “knowledge spillovers” have been discussed as an explanation for peer effects among scientists (Waldinger 2010; Azoulay et al. 2010; Borjas and Doran 2012). These articles do not relate to the domain of risk‐taking behavior, however, and do not offer an evolutionary explanation.

31 Some research pursues the idea that risk preferences only change after a long time or when there are exogenous shocks such as the 1929 Great Depression, the 2008 Global Financial Crisis, the 2020 Covid‐19 Pandemic, or war (Malmendier and Nagel 2011; Kim and Lee 2014; Guiso et al. 2018; Tsutsui and Tsutsui‐Kimura 2022).

## Appendix A Proofs

For the reader's convenience, we restate the results here.
Lemma 1 Assume that M is single‐crossing and there are no dominated lotteries in L. Then, for $l^{\prime },l^{\prime \prime }\in L$ with $l^{\prime }\neq l^{\prime \prime }$, exactly one of the following statements is true:

1. There exists a value $r\left(l^{\prime },l^{\prime \prime }\right)\in \left(\underline{r},\bar{r}\right)$ such that $l^{\prime }{≻}_{r}l^{\prime \prime }$ if $r<r(l^{\prime },l^{\prime \prime })$ and $l^{\prime \prime }{≻}_{r}l^{\prime }$ if $r>r(l^{\prime },l^{\prime \prime })$.
2. There exists a value $r\left(l^{\prime \prime },l^{\prime }\right)\in \left(\underline{r},\bar{r}\right)$ such that $l^{\prime \prime }{≻}_{r}l^{\prime }$ if $r<r(l^{\prime \prime },l^{\prime })$ and $l^{\prime }{≻}_{r}l^{\prime \prime }$ if $r>r(l^{\prime \prime },l^{\prime })$.

Consider the sets

$$A:=\left\{\;r\in \left(\underline{r},\bar{r}\right)\;|\;l^{\prime }{≻}_{r}l^{\prime \prime }\;\right\}\;\text{and}\;B:=\left\{\;r\in \left(\underline{r},\bar{r}\right)\;|\;l^{\prime \prime }{≻}_{r}l^{\prime }\;\right\}.$$

If A was empty, then lottery $l^{\prime }$ was dominated, and if B was empty, then $l^{\prime \prime }$ was dominated. Therefore, both sets are nonempty. Because ${≻}_{r}$ is a complete order for all r, it holds $A\cup B=(\underline{r},\bar{r})$. From this follows that $\underline{r}=\inf A$ or $\underline{r}=\inf B$. If neither was the case, we would have $\inf A,\inf B>\underline{r}$, and then, by definition of the infimum, any value r with $\underline{r}<r<\min\left[\inf A,\inf B\right]$ would be neither in A nor in B, contradicting $A\cup B=(\underline{r},\bar{r})$. Assume $\underline{r}=\inf A$ (this does not lose generality as our argument can be made analogously for $\underline{r}=\inf B$). Pick $r^{\prime }\in A$ and $r^{\prime \prime }\in B$. $r^{\prime \prime }<r^{\prime }$ is impossible because the single‐crossing property would require that for all $r<r^{\prime \prime }$ holds r∈B, but then $\inf A\geq r^{\prime \prime }>\underline{r}$, contrary to our assumption. Therefore, we have $r^{\prime }<r^{\prime \prime }$. As we chose $r^{\prime },r^{\prime \prime }$ arbitrarily from A and B, this implies that supA≤infB. However, there exists no value r with supA<r<infB because such r would be neither in A nor in B. The case supA<infB is thus impossible, and it follows that we must have supA=infB. Then, $r(l^{\prime },l^{\prime \prime }):=\sup A=\inf B$ has the desired properties by the definition of the supremum, the infimum, and the sets A and B. Our argument was based on the assumption $\underline{r}=\inf A$. If we would assume $\underline{r}=\inf B$ instead, the argument can be made analogously and it follows that supB=infA, and $r(l^{\prime \prime },l^{\prime }):=\sup B=\inf A$ has the desired properties. It remains to be shown that exactly one of the two cases, either $\underline{r}=\inf A$ or $\underline{r}=\inf B$, is true. Pick $r^{\prime },r^{\prime \prime }$ such that $r^{\prime }\in A$ and $r^{\prime \prime }\in B$. Trivially, either $r^{\prime }>r^{\prime \prime }$ or $r^{\prime \prime }>r^{\prime }$ is true. If $r^{\prime }>r^{\prime \prime }$, the single‐crossing property requires that for every $r\leq r^{\prime \prime }$, it holds r∈B; hence, $\underline{r}\neq \inf A$. Analogously, from $r^{\prime \prime }>r^{\prime }$ follows $\underline{r}\neq \inf B$.□

Lemma 2 The function d:L×L→N∪{0} defined by (1) is a metric on L.

It is clear that $d({≻}^{\prime },{≻}^{\prime })=0$ and $d\left({≻}^{\prime },{≻}^{\prime \prime }\right)=d\left({≻}^{\prime \prime },{≻}^{\prime }\right)>0$ for ${≻}^{\prime },{≻}^{\prime \prime }\;\in \;P$ with ${≻}^{\prime }\;\neq \;{≻}^{\prime \prime }$. Only the Triangle inequality

(A.1)
$$d\left({≻}^{\prime },{≻}^{\prime \prime \prime }\right)\leq d\left({≻}^{\prime },{≻}^{\prime \prime }\right)+d\left({≻}^{\prime \prime },{≻}^{\prime \prime \prime }\right)$$

requires an argument. Here, we note that for each two unequal lotteries that are ranked differently by ${≻}^{\prime }$ and ${≻}^{\prime \prime \prime }$, the relation ${≻}^{\prime \prime }$ either agrees with ${≻}^{\prime }$ or with ${≻}^{\prime \prime \prime }$ because there are three relations but, in the absence of indifference, only two ways how to rank the two elements. Specifically, consider a pair $(l^{\prime },l^{\prime \prime })\in L\times L$ that is counted for the value of $d({≻}^{\prime },{≻}^{\prime \prime \prime })$, that is, for the left‐hand side of (A.1). Then, if ${≻}^{\prime \prime }$ agrees with ${≻}^{\prime }$ on $\left\{l^{\prime },l^{\prime \prime }\right\}$, the pair is not counted for $d({≻}^{\prime },{≻}^{\prime \prime })$. However, it is then counted for $d({≻}^{\prime \prime },{≻}^{\prime \prime \prime })$. Thus, this pair is also counted once for the right‐hand side of (A.1). Alternatively, if ${≻}^{\prime \prime }$ agrees with ${≻}^{\prime \prime \prime }$ on $\left\{l^{\prime },l^{\prime \prime }\right\}$, the pair is counted for $d({≻}^{\prime },{≻}^{\prime \prime })$ but not for $d({≻}^{\prime \prime },{≻}^{\prime \prime \prime })$. Again, the pair is also counted once for the right‐hand side of (A.1). Hence, if we restrict the relations to the subset of pairs in L×L which are counted for $d({≻}^{\prime },{≻}^{\prime \prime \prime })$, the Triangle inequality is fulfilled with equality. Among those pairs that are ranked equally by ${≻}^{\prime }$ and ${≻}^{\prime \prime \prime }$, that is, which are not counted for $d({≻}^{\prime },{≻}^{\prime \prime \prime })$, some may be ranked differently by ${≻}^{\prime \prime }$ and by ${≻}^{\prime }$ and ${≻}^{\prime \prime \prime }$. These would be counted for both $d({≻}^{\prime },{≻}^{\prime \prime })$ and $d({≻}^{\prime \prime },{≻}^{\prime \prime \prime })$, raising the right‐hand side of (A.1) above its left‐hand side. In the presence of such pairs, the Triangle inequality is fulfilled strictly.□

Lemma 3 Let $\{l^{1},l^{2},l^{3},l^{4}\}\in L$ with $\left\{l^{1},l^{2}\right\}\neq \left\{l^{3},l^{4}\right\}$. If the thrive function M is single‐crossing and linear, then $r\left(l^{1},l^{2}\right)\neq r\left(l^{3},l^{4}\right)$.

Let $R:=\{\;r\left(l^{\prime },l^{\prime \prime }\right)\in \left(\underline{r},\bar{r}\right)\;|\;l^{\prime },l^{\prime \prime }\in L\;\}$ be the set of values whose existence was proved in Lemma 1. By way of contradiction, assume that there are lotteries $\left\{l^{1},l^{2}\right\}\neq \left\{l^{3},l^{4}\right\}$ with $\hat{r}:=r\left(l^{1},l^{2}\right)=r\left(l^{3},l^{4}\right)$. Set

$$a:=\max\left\{x\in \left\{\underline{r}\right\}\cup R\;|\;x<\hat{r}\right\}\;\text{and}\;b:=\min\left\{x\in \left\{\bar{r}\right\}\cup R\;|\;x>\hat{r}\right\}.$$

Select $r^{\prime }\in \left(a,\hat{r}\right)$ and $r^{\prime \prime }\in \left(\hat{r},b\right)$. Then, $d({≻}_{r^{\prime }},{≻}_{r^{\prime \prime }})>2$ because, with $r^{\prime }<\hat{r}$ and $r^{\prime \prime }>\hat{r}$, the relations ${≻}_{r^{\prime }}$ and ${≻}_{r^{\prime \prime }}$ rank (at least) the lotteries $l^{1},l^{2}$ and $l^{3},l^{4}$ differently. Because we assume the thrive function to be linear, there must be values $r^{1},r^{2}$ with $r^{\prime }\leq r^{1}<r^{2}\leq r^{\prime \prime }$ such that for all $r\in (r^{1},r^{2})$, it holds $d({≻}_{r},{≻}_{r^{\prime \prime }})=1$. We will derive a contradiction from here. If there are such values $r^{1},r^{2}$, then $\hat{r}\leq r^{1}$ because left of $\hat{r}$ all ${≻}_{r}$ differ from ${≻}_{r^{\prime \prime }}$ in at least two comparisons. However, for all $r\in (\hat{r},r^{2})$, it holds $d({≻}_{r},{≻}_{r^{\prime \prime }})=0$, that is, the relations ${≻}_{r}$ and ${≻}_{r^{\prime \prime }}$ are the same. To show this, assume again by way of contradiction that there is $r\in (\hat{r},r^{2})$ and two lotteries $l^{\prime },l^{\prime \prime }\in L$ that are ranked differently by ${≻}_{r}$ and ${≻}_{r^{\prime \prime }}$. W.l.o.g., assume $l^{\prime }{≻}_{r}l^{\prime \prime }$ and $l^{\prime \prime }{≻}_{r^{\prime \prime }}l^{\prime }$. By Lemma 1, there exists a value $r(l^{\prime },l^{\prime \prime })$ or a value $r(l^{\prime \prime },l^{\prime })$ with properties as specified in the lemma. Again w.l.o.g., assume the existing value is $r(l^{\prime },l^{\prime \prime })$. If $r\left(l^{\prime },l^{\prime \prime }\right)<r<r^{\prime \prime }$, then, by definition of $r(l^{\prime },l^{\prime \prime })$, we must have $l^{\prime \prime }{≻}_{r}l^{\prime }$, contrary to our assumption. If, on the other hand, we have $r<r^{\prime \prime }<r\left(l^{\prime },l^{\prime \prime }\right)$, then we must have $l^{\prime }{≻}_{r^{\prime \prime }}l^{\prime \prime }$, again contradicting our assumption. It therefore follows that $r\left(l^{\prime },l^{\prime \prime }\right)\in \left[r,r^{\prime \prime }\right]$. Yet, $r(l^{\prime },l^{\prime \prime })$ is in R, and $r^{\prime \prime }$ was picked such that $r^{\prime \prime }<b=\min\left\{x\in \left\{\bar{r}\right\}\cup R\;|\;x>\hat{r}\right\}$, rendering the case $r\left(l^{\prime },l^{\prime \prime }\right)\in \left[r,r^{\prime \prime }\right]\subseteq \left(\hat{r},b\right)$ impossible. This contradiction proves that the existence of lotteries $\left\{l^{1},l^{2}\right\}\neq \left\{l^{3},l^{4}\right\}$ with $\hat{r}:=r\left(l^{1},l^{2}\right)=r\left(l^{3},l^{4}\right)$ is incompatible with linearity.□

Proposition 1 If the function M is single‐crossing and linear, then $\left(\underline{r},\bar{r}\right)$ can be partitioned into n intervals $I_{1},\text{…},I_{n}$ (each of which may be open, closed, or semiclosed) such that:

1. ${≻}_{r^{\prime }}\;=\;{≻}_{r^{\prime \prime }}$ if and only if $r^{\prime },r^{\prime \prime }\in I_{j}$ for one j∈{1,…,n}.
2. The n intervals can be labeled in a way that fulfils:
  $$r^{\prime }\in I_{i},r^{\prime \prime }\in I_{j}\Rightarrow d\left({≻}_{r^{\prime }},{≻}_{r^{\prime \prime }}\right)=\left|i-j\right|.$$

We will carry out the proof by constructing a sequence of labeled intervals and then showing that the intervals and the labeling have the two properties stated above. As before, let $R:=\{\;r\left(l^{\prime },l^{\prime \prime }\right)\in \left(\underline{r},\bar{r}\right)\;|\;l^{\prime },l^{\prime \prime }\in L\;\}$ be the set that contains the values specified in Lemma 1. Define

$$\rho \left(\hat{r}\right):=\left\{\begin{array}{cc} \min\{r\in R\;|\;r>\hat{r}\;\} & \;\text{if}\;\left\{r\in R\;|\;r>\hat{r}\;\right\}\neq \emptyset \bar{r}\;\text{otherwise.} \end{array}\right.$$

With this notation, we devise the following algorithm:
**Initialization**: Set

$$I_{1}:=\left\{\begin{array}{cc} \left(\underline{r},\min R\right] & \;\text{if}\;{≻}_{r}\;=\;{≻}_{\min R}\;\text{for}\;r:=\frac{1}{2}\underline{r}+\frac{1}{2}\min R\\ \left(\underline{r},\min R\right) & \;\text{otherwise.} \end{array}\right.$$

Set j:=2.
**Main iteration**: Step 1: If $\rho \left(\sup I_{j-1}\right)\neq \bar{r}$, go to Step 2. Otherwise, set

$$I_{j}=:\left(\underline{r},\bar{r}\right)\setminus \left\{\underset{k=1,\text{…},j-1}{⋃}I_{k}\right\},$$

terminate the algorithm, and let the output be the intervals $I_{1},\text{…},I_{n}$ with n=j. Step 2: If $I_{j-1}$ is a right open interval, set

$$I_{j}:=\left\{\begin{array}{cc} \left[\text{sup}I_{j-1},\rho \left(\text{sup}I_{j-1}\right)\right] & \text{if}{≻}_{r}\;=\;{≻}_{\rho (\text{sup}I_{j-1})}\text{for}r:=\frac{1}{2}\text{sup}I_{j-1}+\frac{1}{2}\rho \left(\text{sup}I_{j-1}\right)\\ \left[\text{sup}I_{j-1},\rho \left.(\text{sup}I_{j-1})\right)\right. & \text{otherwise.} \end{array}\right.$$

If $I_{j-1}$ is a right closed interval, we set

$$I_{j}:=\left\{\begin{array}{cc} \left(\sup I_{j-1},\rho \left(\sup I_{j-1}\right)\right] & \;\text{if}\;{≻}_{r}\;=\;{≻}_{\rho (\sup I_{j-1})}\;\text{for}\;r:=\frac{1}{2}\sup I_{j-1}+\frac{1}{2}\rho \left(\sup I_{j-1}\right)\\ \left(\sup I_{j-1},\rho \left(\sup I_{j-1}\right)\right) & \;\text{otherwise.}\; \end{array}\right.$$

Set j:=j+1. Return to Step 1. As there are finitely many elements in L, there are finitely many elements in R. Therefore, there are finitely many steps until $\rho \left(\sup I_{j-1}\right)=\bar{r}$ is reached and the algorithm ends. The first property of the partition, namely, ${≻}_{r^{\prime }}\;=\;{≻}_{r^{\prime \prime }}$ if and only if $r^{\prime },r^{\prime \prime }\in I_{j}$, results from the fact that each interval border (except $\underline{r}$ and $\bar{r}$ of the extreme intervals) is an element of R. For the “if”‐part, consider by way of contradiction that there was an index j with $r^{\prime },r^{\prime \prime }\in I_{j}$ such that $r^{\prime }<r^{\prime \prime }$, and two lotteries $l^{\prime },l^{\prime \prime }$ with $l^{\prime }{≻}_{r^{\prime }}l^{\prime \prime }$ and $l^{\prime \prime }{≻}_{r^{\prime \prime }}l^{\prime }$. By Lemma 1, there must exist a value $r(l^{\prime },l^{\prime \prime })\in R$ or a value $r(l^{\prime \prime },l^{\prime })\in R$. W.l.o.g., assume $r(l^{\prime },l^{\prime \prime })$ exists. By definition of $r(l^{\prime },l^{\prime \prime })$, if $r\left(l^{\prime },l^{\prime \prime }\right)<r^{\prime }$, then $l^{\prime }{≻}_{r^{\prime }}l^{\prime \prime }$ is impossible. Likewise, if $r\left(l^{\prime },l^{\prime \prime }\right)>r^{\prime \prime }$, then $l^{\prime \prime }{≻}_{r^{\prime \prime }}l^{\prime }$ is impossible. Therefore, we must have $r^{\prime }\leq r\left(l^{\prime },l^{\prime \prime }\right)\leq r^{\prime \prime }$. As $r(l^{\prime },l^{\prime \prime })\in R$, it is in itself an interval border; hence, if $r^{\prime }<r\left(l^{\prime },l^{\prime \prime }\right)<r^{\prime \prime }$, the values $r^{\prime }$ and $r^{\prime \prime }$ could not be from the same interval, contrary to our assumption. If, on the other hand, $r\left(l^{\prime },l^{\prime \prime }\right)=r^{\prime }$ or $r\left(l^{\prime },l^{\prime \prime }\right)=r^{\prime \prime }$, then $r^{\prime }$ or $r^{\prime \prime }$ are themselves boundaries of an interval, and as $r^{\prime },r^{\prime \prime }\in I_{j}$, that interval must be $I_{j}$. W.l.o.g., assume $r\left(l^{\prime },l^{\prime \prime }\right)=r^{\prime }$ is the left boundary of $I_{j}$. That means that $I_{j}$ is left‐closed, which implies $I_{j-1}$ is right‐open. By construction, this is only the case if for the midpoint $\hat{r}$ of $I_{j-1}$ holds ${≻}_{\hat{r}}\;\neq \;{≻}_{r^{\prime }}$. However, by Lemma 3, the only way in which the three relations ${≻}_{\hat{r}}$, ${≻}_{r^{\prime }}$, and ${≻}_{r^{\prime \prime }}$ can differ is by the ranking of $l^{\prime }$ and $l^{\prime \prime }$ (because $r\left(l^{\prime },l^{\prime \prime }\right)=r^{\prime }$, but for any other $l^{1},l^{2}\in L$, we have $r\left(l^{1},l^{2}\right)\neq r^{\prime }$). Thus, as we assume that ${≻}_{r^{\prime }}$ and ${≻}_{r^{\prime \prime }}$ rank $l^{\prime }$ and $l^{\prime \prime }$ differently, it must be the case that ${≻}_{\hat{r}}$ and ${≻}_{r^{\prime }}$ rank $l^{\prime }$ and $l^{\prime \prime }$ in the same way, contradicting that we had already derived ${≻}_{\hat{r}}\;\neq \;{≻}_{r^{\prime }}$. It remains to be shown that if $r^{\prime }\in I_{i},r^{\prime \prime }\in I_{j}\Rightarrow d\left({≻}_{r^{\prime }},{≻}_{r^{\prime \prime }}\right)=\left|i-j\right|$. We can restrict our attention to the case that both $r^{\prime }$ and $r^{\prime \prime }$ are *interior* values in their respective intervals because from the first part of the proof follows that the boundaries of an interval, if they are included in the interval, will be mapped into the same relation as an interior value. Then, considering Lemma 3 and the first part of this proposition, $d({≻}_{r^{\prime }},{≻}_{r^{\prime \prime }})$ equals the number of interval boundaries that are strictly *between* $r^{\prime }$ and $r^{\prime \prime }$. By the way the intervals were constructed and labeled, that is, by defining a sequence of increasing interval boundaries that are labeled consecutively, the number of interval boundaries between the two interior values $r^{\prime }\in I_{i}$ and $r^{\prime \prime }\in I_{j}$ is |i−j|.□

Lemma 4
(Median minimizes expected absolute error) If m(p) is the unique median of the probability mass function p, then i=m(p) minimizes the expression

$$\sum_{k=1,\text{…},\infty }k\left(p\left(i+k\right)+p\left(i-k\right)\right).$$

See, for example, Shanmugam and Chattamvelli (2015), Theorem 2.3 (p. 55).□

Lemma 5 Let p and q be probability mass functions defined on Z, supported on a finite set {1,…,n}⊂N, and let $s_{\lambda }={\left\{\lambda q\left(i\right)+\left(1-\lambda \right)p\left(i\right)\right\}}_{i\in Z}$ with λ∈[0,1] be a linear combination of p and q. If $m\left(q\right),m\left(p\right),m\left(s_{\lambda }\right)$ are the unique medians of the three functions and m(p)≤m(q), then $m\left(p\right)\leq m\left(s_{\lambda }\right)\leq m\left(q\right)$.

By definition, $m(s_{\lambda })$ is the smallest index $\bar{m}$ for which

(A.2)
$$\lambda \sum_{k=1,\text{…},\bar{m}}p\left(k\right)+\left(1-\lambda \right)\sum_{j=1,\text{…},\bar{m}}q\left(j\right)\geq 0.5$$

If $\bar{m}<m\left(p\right)$, we have $\sum_{k=1,\text{…},\bar{m}}p\left(k\right)<0.5$ and, because of m(p)≤m(q), $\sum_{j=1,\text{…},\bar{m}}q\left(j\right)<0.5$, and thus,

$$\lambda \underset{<0.5}{\underset{︸}{\sum_{k=1,\text{…},\bar{m}}p\left(k\right)}}+\left(1-\lambda \right)\underset{<0.5}{\underset{︸}{\sum_{j=1,\text{…},\bar{m}}q\left(j\right)}}<0.5.$$

Likewise, for $\bar{m}\geq m\left(p\right)$, we have and $\sum_{j=1,\text{…},\bar{m}}q\left(j\right)\geq 0.5$ and, because of m(p)≤m(q), also $\sum_{k=1,\text{…},\bar{m}}p\left(k\right)\geq 0.5$, and thus,

$$\lambda \underset{\geq 0.5}{\underset{︸}{\sum_{k=1,\text{…},\bar{m}}p\left(k\right)}}+\left(1-\lambda \right)\underset{\geq 0.5}{\underset{︸}{\sum_{j=1,\text{…},\bar{m}}q\left(j\right)}}\geq 0.5.$$

Therefore, $\bar{m}>m\left(p\right)$ would not be the smallest index to fulfill (A.2). It follows that we must have $m\left(p\right)\leq m\left(s_{\lambda }\right)\leq m\left(q\right)$.□

Proposition 2 Let λ∈[0,1]. Then,

$$d\left({≻}^{m(s_{\lambda })},{≻}^{m(q)}\right)\leq d\left({≻}^{m(p)},{≻}^{m(q)}\right).$$

If m(p)=m(q), Lemma 5 ensures $m\left(s_{\lambda }\right)=m\left(p\right)=m\left(q\right)$, and thus, in view of Proposition 1 (Statement 1), we have ${≻}^{m(s_{\lambda })}\;=\;{≻}^{m(p)}\;=\;{≻}^{m(q)}$ so that the result is trivially true. W.l.o.g. assume m(p)<m(q). By Proposition 1 (Statement 2), Proposition 2 is true if

(A.3)
$$|m\left(q\right)-m\left(s_{\lambda }\right)|\leq m\left(q\right)-m\left(p\right)$$

(as $m\left(q\right),m\left(p\right),m\left(s_{\lambda }\right)\in \left\{1,\text{…},n\right\}$ and thus $m\left(q\right),m\left(p\right),m\left(s_{\lambda }\right)>0$, we have dropped the absolute value function on the right‐hand side). Because of Lemma 5, there exists δ∈[0,1] such that

(A.4)
$$m\left(s_{\lambda }\right)=\delta m\left(q\right)+\left(1-\delta \right)m\left(p\right).$$

Plugging (A.4) into (A.3) leads to

$$\begin{array}{c} vertm(q)-\delta m(q)-(1-\delta )m(p)|\leq m(q)-m(p)\Leftrightarrow \\ \left(1-\delta )(m(q)-m(p))\leq m(q)-m(p\right) \end{array}$$

which is obviously true for δ∈[0,1] and 0<m(p)<m(q).□
